# Biotechnological Processes Simulating the Natural Fermentation Process of Bee Bread and Therapeutic Properties—An Overview

**DOI:** 10.3389/fnut.2022.871896

**Published:** 2022-04-27

**Authors:** Daniel Gabriel Barta, Mihaiela Cornea-Cipcigan, Rodica Margaoan, Dan Cristian Vodnar

**Affiliations:** ^1^Institute of Life Sciences, University of Agricultural Sciences and Veterinary Medicine Cluj-Napoca, Cluj-Napoca, Romania; ^2^Faculty of Food Science and Technology, University of Agricultural Sciences and Veterinary Medicine Cluj-Napoca, Cluj-Napoca, Romania; ^3^Advanced Horticultural Research Institute of Transylvania, University of Agricultural Sciences and Veterinary Medicine Cluj-Napoca, Cluj-Napoca, Romania

**Keywords:** bee bread, lactic acid bacteria, fermented product, food supplement, a probiotic product

## Abstract

Recent signs of progress in functional foods and nutraceuticals highlighted the favorable impact of bioactive molecules on human health and longevity. As an outcome of the fermentation process, an increasing interest is developed in bee products. Bee bread (BB) is a different product intended for humans and bees, resulting from bee pollen's lactic fermentation in the honeycombs, abundant in polyphenols, nutrients (vitamins and proteins), fatty acids, and minerals. BB conservation is correlated to bacteria metabolites, mainly created by *Pseudomonas* spp., *Lactobacillus* spp., and *Saccharomyces* spp., which give lactic acid bacteria the ability to outperform other microbial groups. Because of enzymatic transformations, the fermentation process increases the content of new compounds. After the fermentation process is finalized, the meaningful content of lactic acid and several metabolites prevent the damage caused by various pathogens that could influence the quality of BB. Over the last few years, there has been an increase in bee pollen fermentation processes to unconventional dietary and functional supplements. The use of the chosen starters improves the bioavailability and digestibility of bioactive substances naturally found in bee pollen. As a consequence of enzymatic changes, the fermentation process enhances BB components and preserves them against loss of characteristics. In this aspect, the present review describes the current biotechnological advancements in the development of BB rich in beneficial components derived from bee pollen fermentation and its use as a food supplement and probiotic product with increased shelf life and multiple health benefits.

## Introduction

Nowadays, the concept of “food as medicine and medicine as food” assimilated to Hippocrates is increasingly common in the food sector, where the production of functional foods is a significant part of human lifestyle (1). All over the world, consumers have extensive knowledge regarding the effect of food on wellbeing and having positive food expectations (2).

Bee products have been used to treat and prevent disorders such as burns, wounds, diabetic foot ulcers, allergic rhinitis, hyperlipidemia, and rheumatoid arthritis through the history of the traditional medicine (3–6). Recently, bee products developed due to the fermentation process have gained colossal interest and became a severe topic for future research due to the positive impact on the food market.

Bee bread (BB) is a fermented assortment of bee pollen (BP), nectar, and bee saliva, having a caramel-like color and a sharp taste given by flowers, citrus, or other fruit flavors (7). The outcome of lactic acid fermentation of BP collected from flowers by bees and combined with their digestive enzymes is the primary food for larvae and young bees in the hive (8). In the cells of the honeycombs, bees pack the components and then secure the mixture with honey and wax, protecting the pollen mass from oxygen, starting an anaerobic lactic fermentation process, which after approximately seven days generates BB (9).

According to Habryka et al. BB incorporates a well-balanced nutritional content and more prosperous chemical composition than BP, having a significant assimilation rate and a better absorption at the level of the human body (10). Since the BB components are fermented in part, the high BP content is more straightforward incorporated and utilized as the walls have partly deteriorated through the lactic fermentation (9). The studies on its chemical composition demonstrated that BB is a trustworthy source of proteins, vitamins (B, C, E, K, and P), and polyphenols, like quercetin, kaempferol, apigenin, naringenin, chrysin, caffeic, gallic and ferulic acids (11). Besides, free amino acids, carbohydrates, and fatty acids are essential elements of BB composition, which depend on environmental conditions, seasonal discrepancy and differ from region to region, based on the melliferous plant's (12).

The recent studies on BB chemical composition established that it has a higher nutritional value than BP; nevertheless, research on BB is scarce and focused only on its chemical composition (13, 14). Because collecting BB from the hive is too costly and time-consuming, BB purchases for human consumption are limited; moreover, beekeepers feel more comfortable extracting BP using traps. Unfortunately, due to this method, there is a danger of harmful mold growth due to the high humidity. Furthermore, storage treatments are required to avoid spoiling (15–17). The traditional methods used have several disadvantages; the nutritional value is affected, and the operating cost is high; therefore, alternative opportunities should be considered to overcome these impediments. A unique opportunity is the biotechnological fermentation of BP, simulating the natural process in the hive leading to a high nutritional product, as in BB (18).

Fermentation is one of the most widely used methods in the production and economic preservation of food, being perceived as an essential constituent of the nutritional culture of every world's society supporting the cultural history of ethnic communities (19). Globally, a multifariousness of fermented products is widely consumed as daily human food, from yogurt, kefir, sausages to pickles and fermented cereals, thanks to their biological functions and enrichment of nutritional value. In the beehive, the natural fermentation improves the bioavailability of fresh BP and the possibility of long-time storage, avoiding losing its nutritional value as it is converted to BB (20). Because of its beneficial components, BP represents a valuable raw material that allows the microorganisms' development during the fermentation process (21). In the past years, researchers attempted to induce the natural fermentation of BP at a lab scale by inoculating diverse microorganisms under specific process conditions.

The purpose of the review was to present an overview of the biotechnological processes used to obtain BB from harvested BP in laboratory conditions by an assortment of chosen starters reproducing the microbial consortium implicated in the fermentation of BB. Changes in the bioactive compounds and antioxidant activity are also pointed out along with the final's probiotic product shelf life and health benefits. Furthermore, the effectiveness of BB and strains isolated or found in BB are investigated in the prevention and treatment of several anti-cancer agent-induced toxicities in animal models and patients with cancer.

## Similarities and Differences Between Bee Pollen and Bee Bread

Because of their nutritional and medicinal properties, natural products such as unique bee brands have piqued the curiosity of academics in recent years. Even though these have been well-known products for millennia, they have only lately become the subject of recorded scientific investigation (2).

BP is recognized as the oldest nutritive supplement in history and includes roughly all of the dietary nutrition compounds, the main ingredient of BB (14). BP is the male gametophyte of flowering plants. After gathering pollen grains from flowers, bees combine their saliva and secretions. This procedure enables BP to be hydrated and pelleted, which subsequently sticks to the pollen basket on the bees' rear leg and carries to hive. BP is a source of nutrients that honeybees need to grow and develop appropriately throughout their larval stage until maturity (22, 23). BP is deposited into honeycomb cells by bees, which seal them with honey and wax. Collected and stored, BP is exposed to lactic fermentation under the beehive conditions, resulting in BB, which is fermented BP (2).

BP and BB are used for apitherapeutic benefits as they are rich in vitamins, valuable bio-elements, and nutrients. Still nevertheless, the two components differ from a biochemical point of view (Table 1) (25).

**Table 1:** Chemical and nutritious compounds of BP and BB adapted after Kieliszek et al. and Bakour et al. (2, 14, 24).

**Composition**	**BP**	**BB**
Proteins	4.50–40.70%	14–37%
Carbohydrates	24.0–60.0%%	24–74.82%
Lactic acid	0.56%	3.2%
Lipids	1–18%	6–13%
Cellulose	3.7%	2.7%
Nucleic acid	0.6–4.8%	n.a.
pH	3.8–6.3	4.3
Fiber	0.15–31.26%	n.a.
Glucose	13.41/100 g	5.7 ± 0.4
Fructose	15.36 g/100 g	11.8 ± 0.6 g/100 g
Sucrose	4.25 g/100 g	n.a.
Potassium (K)	3.06–13366.6 mg/kg	338 ± 8 mg/100 g
Phosphorus (P)	234.40–9587 mg/kg	251 ± 4 mg/100 g
Calcium (Ca)	1.09–5752.19 mg/kg	198 ± 4 mg/100 g
Magnesium (Mg)	44.0–4680.53 mg/kg	61 ± 2 mg/100 g
Zinc (Zn)	0.1–105.8 mg/kg	3.31 ± 0.04 mg/100 g
Iron (Fe)	2.6–1180.0 mg/kg	27.3 ± 0.3 mg/100 g
Total phenolic content	0.69–213.2 mg GAE/g	9.2 ± 0.1 mg GAE/g

*n.a., no data available; GAE, gallic acid equivalents*.

**Figure 1:**
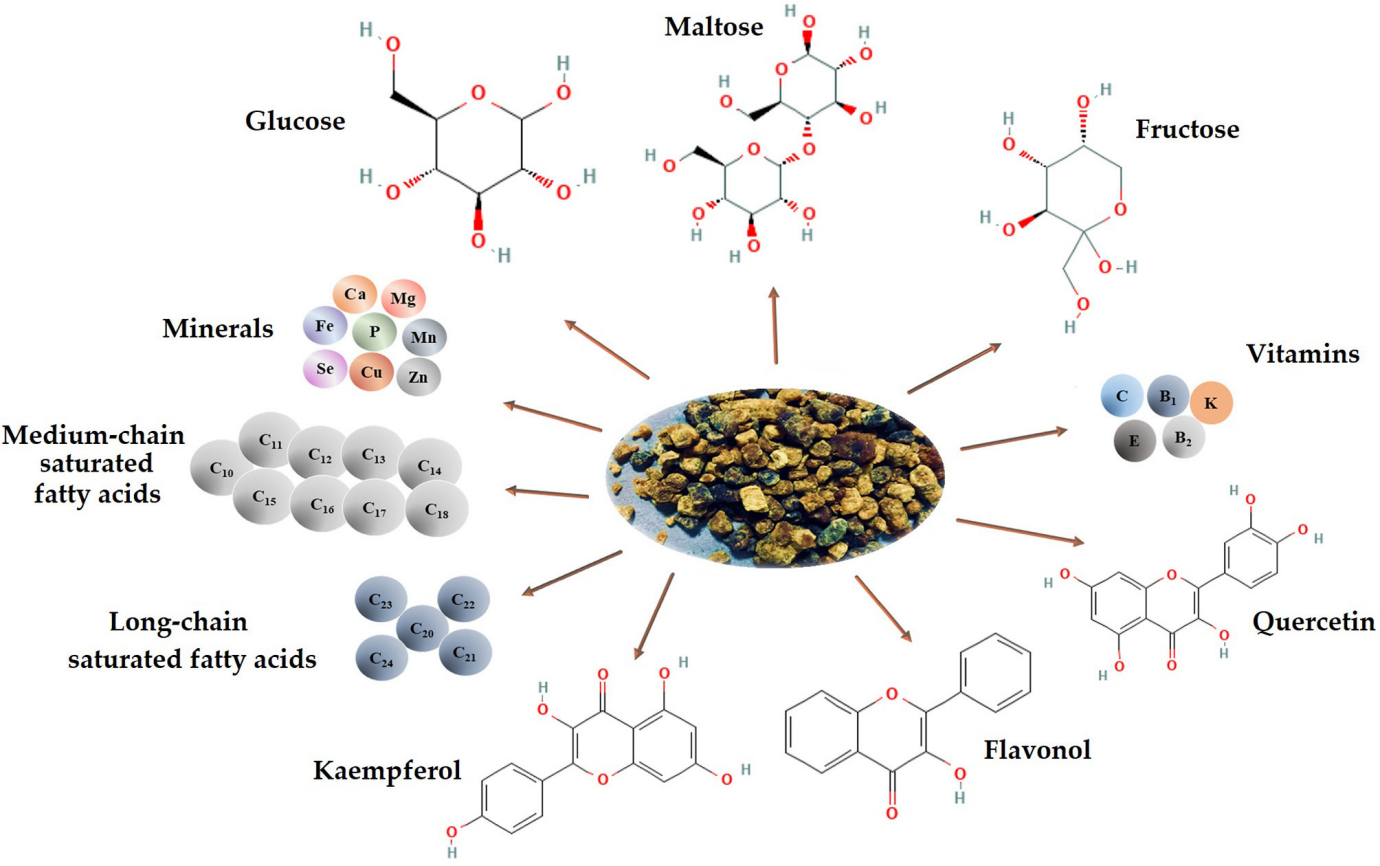
Chemical composition and bioactive compounds of BB (image created using BioRender and King Draw applications).

The nutritional composition of BB varies based on the pollen's local and seasonal value and availability to different plant species. Compared to BP, BB is high in reducing sugars and has significant amounts of tocopherols (26, 27). BB has low protein and fat content but increased carbohydrate and lactic acid content. Due to its structure, it possesses increased bioavailability, which can be partly assimilated by fermentation and demonstrates high resistance against the degradation action of digestive media. Furthermore, the functional and actively rich components of BP are easily absorbed and utilized (Figure 1) (2, 28).

BB is more digestible than BP due to the breakdown of the multilayer wall during fermentation. Moreover, this process enhances BB bioavailability, resulting in higher absorption by human intestinal epithelial cells (3, 18). Also, during fermentation, bacteria break down cellulose, which constitutes the internal coating (also known as intine) of BP, lowering BB's cellulose ratio in contrast to BP (22). Furthermore, throughout the fermentation process, certain innovative products are deliberated. For instance, several proteins in BP are converted to amino acids by digestive enzymes, increasing the protein level, whereas, in BB, an increasing content in amino acids is noticed (2). According to DeGrandi-Hoffman et al., the content of threonine and leucine in BB is approximately 60% higher than BP (29). As a primary food source for bees, BP includes amino acids such as leucine, isoleucine, histidine, lysine, valine, arginine, phenylalanine, methionine threonine, and tryptophan (30). According to Bayram et al., in comparison to BP, BB samples had considerably less L-asparagine (5891.1–2475.4 μg/g), but L-proline (22212.8–4939.2 μg/g), L-aspartic acid (5207.37–2833.3 μg/g), and GABA (4588.4–2703.2 μg/g) were detected as significant amino acids. Furthermore, considerable amounts of L-phenylalanine were found in BP and BB samples, with values ranging from 3353.8–1298.9 μg/g to 3345.6–1308.4 μg/g, respectively (30). The lower content in L-asparagine in BB may be due to the fermentation development and acidic environment that cause this amino acid field's deamination (29).

In 2008, Venskutonis et al. investigated the fatty acid content of BB in summer and spring and discovered 22 fatty acids, as well as five ω-3, four ω-6, and three ω-9 polyunsaturated fatty acids (PUFAs). The primary fatty acids found in BB were arachidonic and oleic acids, with α-linolenic concentrations varying the most between the botanical origins of samples. There was also a substantial difference in the α-linolenic and eicosapentaenoic acids (31).

BP has a high concentration of polyphenolic chemicals, primarily flavonoids and phenolic acids (32). Flavonoids in BP vary from 3.7 to 10.1 mg/g, according to Pascoal et al. (33). At the same time, the total flavonoid content of five BB samples showed values ranging between 13.56 and 18.24 g QE/g DW (quercetin equivalents/g dry weight) (34). Also, in 2015 Zuluaga et al. established that BB originated from Colombia has a total flavonoid content between 1.9 and 4.5 mg QE/g (18).

Vitamins are a diverse group of active ingredients required for the optimal health and growth of all organisms. BB is characterized by higher levels of phenolics with 4.87 mg GAE/g, flavonoids with 59.06 mg QE/g, vitamin C (0.36 ng/g), and E (32.55 ng/g) (35). In addition, K, P, Ca, Mg, Zn, Fe, and Mn (Manganese) levels in spring BB collected from honey bee colonies were also tested at the Institute for Forage Crops, the results demonstrating a strong relationship between the mineral content and the sources of floral pollen (25). Adequate amounts of macro-and microelements in the human organism are critical for the effective operation of many distinct metabolic processes. Minerals are required for appropriate physiological functions and metabolic pathways regulation (36).

BB includes a variety of enzymes, as well as acid leucine aminopeptidase, phosphatase, and glucosidase, which hydrolyze carbohydrates such as amygdalin, salicin, cellobiose, and centipoise (3). The BB fermentation technique results in higher amounts of lactic acid due to microbial metabolism, which provides long-term defense against pathogens while also increasing the nutritious qualities of BB (3).

In summary, BB includes a wide range of nutritional phytochemicals with valuable characteristics, including vitamins, carotenoids, phenolic acids, and, most notably, flavonoids. BB was found to possess antioxidant, antibacterial, antiviral, anti-inflammatory, and anti-cancer benefits because of their complex chemical composition. In recent years, there has been a surge in interest in the chemicals responsible for these effects; hence, taking into account food productions and the beneficial impacts on human wellbeing, these bee products, with enormous production perspectives and usage as natural and valuable components, provide a broad topic of study (37–41).

The growing global interest in functional products and rising health consciousness adds burden to the upcoming production of bee products. BB has the advantage, apart from a distinct chemical profile, the further production of microbial fermentation, which contributes to its nutritional and health properties. BB is quickly absorbed and digested and contains a variety of macro-and micronutrients that are beneficial to the human body, including flavonoids and polyphenols (3). In addition to the well-known bee products, such as BP, honey, and royal jelly, BB might be the potentially exploited gold mine in the culinary sector and medicine development.

## BB Natural Fermentation Process

After fermentation of BP, the resulting product (BB) has an amber-like color and a rich aroma of flowers and citrus flavor (7). The digestive enzymes of bees naturally contain lactic acid bacteria (LAB), due to which a lactic fermentation begins, leading to BB (15, 22, 29).

**Figure 2:**
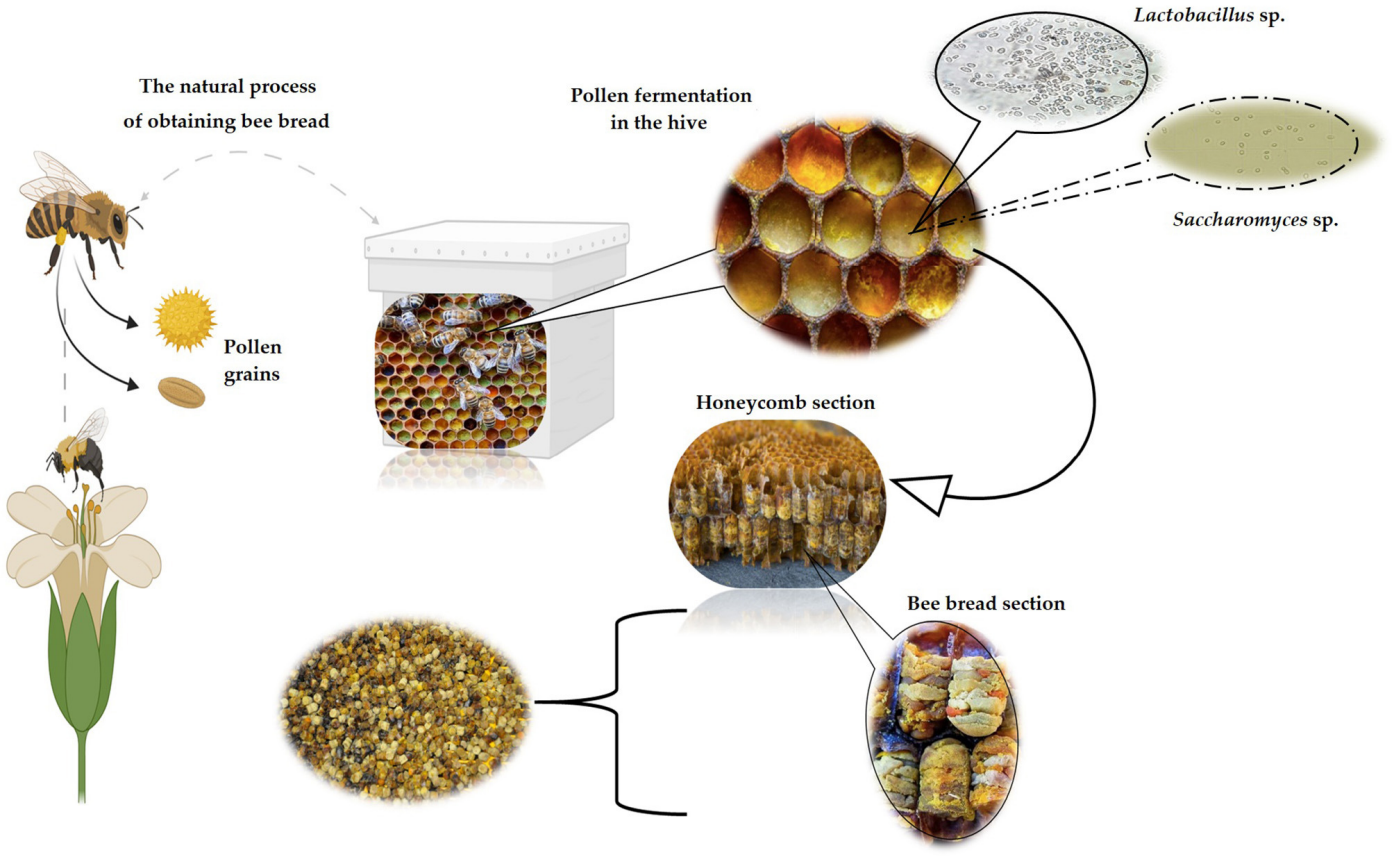
The natural process of producing BB by bees (image created using BioRender and King Draw applications).

According to the indoor beehive conditions, the process of obtaining BB is based on the progression of the appearance/disappearance of colonizing microorganisms, particularly LAB, under anaerobic conditions (18). Also, through the saliva of bees, enzymes are segregated, causing fermentation and enzymatic processes, biochemical transformations necessary to break the outer layer known as exine, that covers the pollen, consisting of sporopollenin that provides resistance to chemicals and preservation of bioactive substances inside the pollen grain (42). As a result of microbial metabolism and biochemical changes, BP is transformed to BB *via* lactic acid fermentation generated mainly by bacteria, like *Pseudomonas* spp., *Lactobacilli* spp., and yeasts as *Saccharomyces* spp. (Figure 2).

In addition, *Bacillus* species in *Melipona panamica* nests were shown to secrete enzymes that catalyze the breakdown of lipids, carbohydrates, and proteins. This bacterial genus was found predominant in BP, and some species are known to ferment glucose. Therefore, this suggests that Bacillus species is a significant genus that could be involved in the BB formation (43, 44).

Inside the hive, BP turns into BB in about 7 days, fulfilling several biochemical stages, starting after the development of LAB, indole-producing bacteria (*Escherichia* spp.), aerobic bacteria, and yeast. In the next step, the anaerobic LAB (*Streptococcus* spp.) use the nutrients created by bacteria and yeast, causing the pH of BP to decrease. Then, when the concentration of *Streptococcus* bacteria decreases, *Lactobacilli* bacteria begin to increase at the end of the 7 days when the LAB and yeasts die. Moreover, due to lactic acid fermentation, the BB reaches a pH of 4.0, becoming microbiologically sterile, all in the last stage (3). Certainly, BB is pollen stored in the hive, which undergoes a fermentation process, most likely due to the glandular secretions of bees and its microbial group, involving mold, yeast, and bacteria, and LAB with a critical role in this process (45). The resulted product is more stable and more nutritious than fresh BP and has higher vitamin content, especially vitamin K because of the pollen degradation (3, 46). Due to the metabolism of microorganisms involved in the fermentation process, the content of LAB increases, resulting in long-term protection against microorganisms and strengthening the nutritional properties of BB (47).

The chemical composition of BB, such as flavor, color, and texture, changes considerably after being stored, and these characteristics vary among bee species. For example, the BB of stingless bee *Frieseomelitta* and *Tetragonisca* are dry and sweet, while the ones produced by *Melipona* and *Scaptotrigona* are moist and sour (48).

The process of collecting BB from the hive is more laborious than BP, where the traps placed at the hive entrance are sufficient to manage it. Complex techniques like machinery separation or freezing followed by a manual crushing of the combs are required to obtain BB as it is fixed with beeswax and tightly stuffed in the combs by bees (22). Therefore, BB is more costly than other bee products (49).

The current tendency relating to the consumer's behavior and food inclination determined the production of novel products. Therefore, new technologies for their development, challenging companies from several food enterprises to commence the action of organic products. As a result of fermentation, the obtained bee products have become an increasingly studied niche. Solid-state fermentation is a bioprocess naturally produced in the hive when BP is used as the primary nutrient source for microorganisms (21). The advantage of using BP in future processes, like solid-state fermentation, will be discussed in the following section to demonstrate the necessity of obtaining BB via the biotechnological route.

## Biotechnological Processes Simulating the Natural Fermentation Process of BB

Nowadays, more and more scientific research proposes using LAB as a noteworthy part of future production chains. The food industries focused on producing value-added products with significant bio-elements, macro and micro-nutrients, vitamins, and health benefits (50). LAB are gram-positive bacteria, non-spore-forming, fermentative, facultative anaerobic, with a significant impact on the food industry (51). Furthermore, LAB has important significance since they fulfill the safety conditions for people and animals (GRAS—-Generally Recognized as Safe). Each has a distinct fermentation metabolism and energy gained due to the saccharides conversion (52).

**Figure 3:**
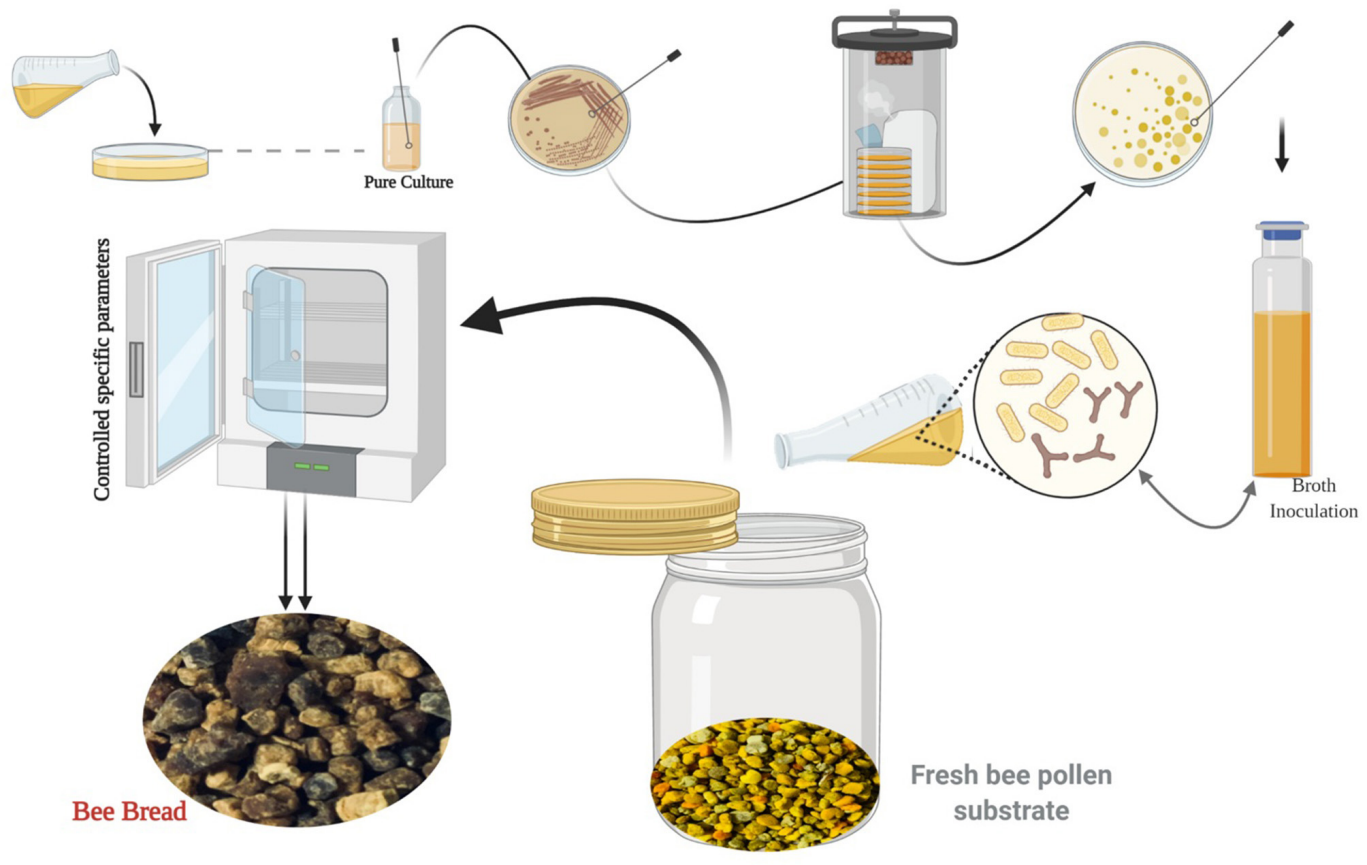
The biotechnological process of simulating the natural fermentation process of BB (image created using BioRender and King Draw applications).

In consequence, throughout fermentation, in addition to lactic acid, the specific LAB strains produce multiple metabolites like exopolysaccharides, enzymes, diacetyl, hydrogen peroxide, and bactericidal proteins or bacteriocins. These compounds confer the functional properties of LAB, for instance, probiotic and fibrinolytic effects antioxidant activity, in addition to providing fermented products with their remarkable consistency, color, flavor, and aroma (53). LAB has a long history of application in various industrial sectors used as starter cultures. Thus, microbial preparations of many microorganisms are introduced in raw material to develop a fermented product by an accelerated and guided process (54, 55). In recent years, numerous researchers attempted to simulate the natural fermentation of BP *via* microorganisms' inoculation under various process conditions at the lab scale to obtain nutritional and functional BB (Figure 3).

Moreover, other studies have carried out enzymatic hydrolysis and sonication as alternative methods to improve BP nutrient bioavailability (56). Concerning LAB, 45 bacteria species were identified and isolated from honeybees, flowers, and bee products, as shown in Table 2 (87). Studies have shown that bees possess specific microbiota, different from other beings, but closely related to the thread Firmicutes, Actinobacteria, and Proteobacteria, the essential intestinal bacteria (88). A unique LAB group, different from the classic consortium that prefers glucose, has been identified in the stomach of bees, fructophilic lactic acid bacteria (FLAB), which can use fructose from richer sources, for instance, flowers (57). Recently, FLAB was regarded as unconventional LAB, as they have a unique growth characteristic due to a partially bi-functional alcohol/acetaldehyde dehydrogenase encoded gene, generating a disequilibrium in NAD/NADH (nicotinamide adenine dinucleotide/reduced nicotinamide adenine dinucleotide) and the necessity of supplementary acceptors for metabolizing glucose (58). According to recent studies, fructose-feeding insects, like bees, possess in their guts high amounts of FLAB cells belonging to the *Fructobacillus* and *Lactobacilli* spp. (59).

**Table 2:** LAB from the colony environment of honeybees.

**Host specie**	**Primary location**	**Isolated microbial strains**	**References**
**Honeybee**			
*Apis* and *Bombus* spp.	Adult hindgut (rectum)	*B. asteriodes*	(57–60)
	Adult hindgut (rectum)	*B. coryneforme*	(57)
	Midgut	*B. indicum*	(61, 62)
	Adult crop	*Bifidobacterium spp*.	(63)
	Adult crop, larval gut, not present in adult hindgut	*Apilactobacillus kunkeei*	(56, 57, 59, 61, 63–66)
	Adult hindgut (rectum)	*Lactobacillus johnsonii*	(59, 67)
	Adult hindgut (rectum)	*Lactiplantibacillus plantarum*	(59, 68)
	Larval gut, adult crop	*Apilactobacillus apinorum*	(69, 70)
	Adult hindgut (rectum)	*Lactobacillus mellis*	(69)
	Adult crop, midgut and rectum	*A. kunkeei*	(56, 57, 59, 61, 63–66)
*Apis cerana* and *A. cerana indica*	Midgut	*Lactobacillus kullabergensis, Bifidobacterium longum*	(59, 69, 71)
*A. dorsata*	Adult crop	*Bombilactobacillus mellifer, Lactobacillus insectis, Enterococcus durans, Oenococcus spp*.	(56, 69, 72, 73)
*A. florea*	Adult hindgut (rectum)	*Enterococcus faecium*	(67)
*A. mellifera* and *A. mellifera* Buckfast	Adult crop	*L. kullabergensis*	(59, 69)
	Adult crop and hindgut	*B. mellifer*	(69)
	Adult crop, midgut and rectum	*Lactobacillus melliventris*	(69)
*A. mellifera*	Adult crop	*Lactobacillus apis, Lactobacillus acidophilus, Lactobacillus alvei, Lentilactobacillus buchneri*	(56, 74, 75)
	Adult crop, midgut and rectum	*Lactobacillus helsingborgensis, Lactobacillus kimbaldii*	(59, 69)
	Midgut	*Lactobacillus brevis*	(61)
	Adult gut, variably present	*Alpha 1*	(58)
	Adult gut	*Fructobacillus pseudoficulneus, Fructobacillus tropaeoli*	(72, 76)
*A. mellifera intermissa*	Adult hindgut (rectum)	*Enterococcus faecalis*	(63)
*A. mellifera, Bombus terrestris, Osmia bicornis* (red mason bee)	Adult gut	*Lactobacillus intestinalis*	(74)
*A. mellifera, Heterotrigona itama, Bombus* spp.	Adult gut	*Fructobacillus fructosus*	(76)
**Honey**			
*Apis* and *Bombus* spp.		*A. kunkeei, L. acidophilus, Lactobacillus crispatus ST1, Furfurilactobacillus rossiae, Companilactobacillus versmoldensis, Ligilactobacillus araffinosus*	(56, 57, 71, 77, 78)
		*Bifidobacterium steroids, Bifidobacterium catenulatum, Bifidobacterium longum*	(59, 60, 71)
*H. itama*		*F. fructosus*	(76)
**BP**			
*Apis* and *Bombus* spp.		*Lactobacillus kefiranofaciens*	(56)
		*Holzapfelia floricola, Apilactobacillus ozensis, Lactobacillus frumenti*	(56)
*A. dorsata*		*L. insectis*	(56)
*A. florea*		*Lactobacillus helsingborgensis*	(69)
*A. mellifera*		*A. kunkeei, L. plantarum, Apilactobacillus apinorum, Lactobacillus alvei, Latilactobacillus curvatus*	(45, 56)
*A. mellifera intermissa*		*E. faecalis*	(79)
*A. mellifera, B. terrestris*		*L. intestinalis, Limosilactobacillus reuteri, Lactococcus lactis*	(56, 80)
*Meliponula bocandei*		*Lactobacillus kimbaldii, Lactobacillus kullabergensis*	(69)
**Royal jelly**			
*A. dorsata* and *A. mellifera*		*A. kunkeei, L. insectis, Bifidobacterium* spp.	(56)
**BB**			
*A. dorsata*		*E. durans, Oenococcus* spp.	(72, 73)
*A. mellifera*		*A. kunkeei, L. plantarum, A apinorum, L. helsingborgensis, Weissella paramesenteroides; Zygosaccharomyces favi spp. nov*	(56, 63, 69, 72, 81, 82)
*A. mellifera lingustica*		*A. kunkeei, L. plantarum, F. fructosus, F.fructosus* JCM 1119 and NBRC 3516, *Levilactobacillus brevis (Lvb. brevis) and Lactobacillus delbrueckii subsp. lactis*	(83, 84)
*A. mellifera, B. terrestris*		*Limosilactobacillus mucosae, Bifidobacterium spp*.	(56)
*H. itama*		*Lactobacillus* spp., *Carnimonas* spp., *Escherichia-Shigella* and *Acinetobacter* spp.	(85)
*M. bocandei*		*Lactobacillus kimbladii, L. kullabergensis*	(69)
Stingless bee *Tetragonula pagdeni*		*Bacillus spp., Streptomyces spp*.	(86)
**Flowers**			
*Paeonia suffruticosa* and *Chrysanthemoides monilifera*		*Fructilactobacillus florum*	(70)
*Hedera helix*		*Lactobacillus lactis*	(45)
Mountain flowers		*Apilactobacillus ozensis*	(56)
*Acacia* spp. And *Mesquite* spp.		*Weissella* spp.	(63)

Foods fermented with selected *Bifidobacterium* strains are known to present a variety of probiotics, prophylactic and therapeutic properties (89). Members of the *Bifidobacterium* genus are found as gut microbiota components. They are thought to play a significant function in sustaining and improving human health by evoking various positive qualities. *Bifidobacteria* may use a wide variety of dietary carbohydrates, most of them being oligo- and polysaccharides derived from plants, that avoid decomposing in the intestine's upper section (90). *Bifidobacteria* spp. are Gram-positive, hetero-fermentative microorganisms that do not generate spores (90). The *Bifidobacteria* pathway consists of a carbohydrate metabolism mechanism found in all *Bifidobacterium* spp., due to their ability to metabolize different polysaccharides leading to the degradation of those undigested sugars (91). Vamanu et al. (92) used a substrate of prebiotics such as lactulose, inulin, and raffinose with *Bifidobacterium bifidum* 1 and 2 to evaluate their impact on lactic acid production, cell viability, and antioxidant activity. The BP and honey-based medium supplemented with prebiotics, ground pollen, and inulin provided the best results in viability and total antioxidant activity (92). These findings demonstrate the feasibility of BB functional foods that can be used in both medicine and food sectors (93).

### *Lactobacilli* spp. on the Biotechnological Process and the Use of Probiotics

Lactobacilli are gram-positive, non-spore-forming bacteria that at the end of fermentation release lactic acid as the primary end product of fermentation, which also contributes to the texture and sensory profile of a food (94). Lactobacillus was the most numerous genera within the LAB group belonging to the phylum Firmicutes, class Bacilli, order II Lactobacillales and family Lactobacillaceae. According to the recent reclassification, the Lactobacillus genus has been split into 23 new genera (95). Some of them (for example, *Lacticaseibacillus casei* and *Ligilactobacillus salivarius*) provide particularly L (+) lactic acid, while other microorganisms like *Lactobacillus bulgaricus* and *Lactobacillus jensenii* generate only D – lactic acid, and microorganisms like *Lactobacillus acidophilus* and *Lactobacillus helveticus* can create a combination of D (+) and L– lactic acid. (63). The live microorganism belonging to these genera has recently gained further attention. They are immune to gastrointestinal acidity, improve the intestinal microbiota, and reduce the growth of undesirable bacteria (65). Further, the available studies in the literature describe a biotechnological way to obtain BB by using collected BP as substrate and specific *Lactobacilli* spp. as starter culture according to their particular growing conditions (Table 3).

**Table 3:** Recent advances in the biotechnological processes to obtain fermented products using BP.

**Floral sample**	**Fermented product**	**Fermentation methods**	**Substrate formulation**	**Fermentation conditions**	**Optimum fermentation conditions and observations**	**References**
*Hedera helix* BP	Fermented BP and BB	Assorted inoculum of *A. kunkeei* strains and *H. uvarum* in fresh BP	Water:pollen (1:4)	30°C for 216 h	Optimal fermentation: unstarted BP; ↑ mesophilic microorganisms and LAB after 40 days; ↑ yeasts afer 20 days in started and unstarted BP; ↓ yeasts in raw BP after 20 days; ↑ protein digestibility in started BP after 216 h at 30°C; ↑ free aminoacids in started and unstarted BP; RawBP and unstarted BP populated by *A. parasiticus* following 10 days of storage; 15 days of hive-storage: *L. plantarum, A. kunkeei, Latilactobacillus curvatus, Leuconostoc citreum, Lactococcus lactis, F. fructosus*; 30 days of hive-storage: *A. kunkeei* and *F. fructosus*	(45)
*Hedera helix* BP	Started-BP; Unstarted-BP; Raw-BP	Comparison between BP fermented with selected strains (Started-BP), spontaneously fermented BP (Unstarted-BP) and unprocessed raw BP (Raw-BP).	Water:pollen (1:4)	30°C for 216 h	↑ bioaccessible phenolics in started BP compared to raw BP; ↑ volatile free fatty acids and acetic acid content in Unstarted-BP	(66)
*Brassica campestris* L. BP from China	Fermented BP	*Lactobacillus delbrueckii* subsp. *bulgaricus, St. thermophilus*and active dry yeast	Water:pollen ratio (1:1)	50 g of BP/WBP mixtures combined with 3% LAB, fermented for 48 h at 42°C; 50 g of BP/WBP mixtures combined with yeast 3%, fermented for 48 h at 37°C; 50 g of BP/WBP mixtures combined with 3% LAB and 3% yeast, fermented for 48 h at 37°C	↑ PUFA, FA and aminoacids in yeast fermented BP; Alpha 2.2 bacteria, *A. kunkeei, Actinobacteria* in unstarted-BP	(96)
*Brassica napus* BP from Slovakia	Fermented pollen cans	Without selected strains	Water (75 ml), honey (45 g) and BP (300 g)	1^st^ Fermentation: V1: 30°C for 2 days, no O_2_ V2: 23°C for 5 days, O_2_ V3: 30 °C for 2 days, no O_2_ 2^nd^ Fermentation: V1: 23 °C for 16 days, no O_2_ V2: 23 °C for 13 days, no O_2_ V3: 23 °C for 6 days, no O_2_	↓ filamentous microscopic fungi by fermentation	(56)
*Pinus* spp. BP	Fermented BP, yeast-fermented BP	*Lacticaseibacillus paracasei* Lc-3	200 mL medium after inoculation with 5–15% (v/v) of starter culture	35–45°C on a shaker for 1–5 day	Inoculum size: 11.92%, at 39.6°C, and pH = 7.22; Viable count = 4.24 × 109 CFU/mL and crude protein = 15.35%	(77)
*Pinus* spp. BP from China	Fermented BP	*L. paracasei* Lc-3; Isolated and characterized strain of *Bacillus coagulans*	200 mL medium after inoculation with 5–15% (v/v) of the starter culture	35–55°C on a shaker for 6–72 h	Optimum viable count production: inoculum size = 9.22%, 49.21°C, and pH = 6.82; treatment of fermented products was carried out by spray-drying	(72)
BP (Apis mellifera) from Colombia	Fermented BP	S1: *L. delbrueckii ssp. bulgaricus, ssp. lactis*, and *St. thermophilus;* S2: *L. delbrueckii ssp. lactis, ssp. cremoris, and ssp. biovar. diacetylactis*); S3: *L. acidophilus* NCFM	Water:BP ratio (1:1 and 2:1), temperature of 115°C between 10 and 20 min; pH = 5.8	72 h at 37°C	Fermented BP with *L. acidophilus* considered as optimum and probiotic; Optimal conditions in 2:1 water:BP mixture with pH = 5.8, heat/pressure pre-treatment for 10 min; at 115°C	(67)
*Hypochaeris* spp. and *Brassica* spp. BP from Colombia	Fermented BP (probiotic characterized product)	S1: fresh BP, S2: BB and BP fermented with Choozit®, S3: *L. plantarum*, S4: *S. cerevisiae*, S5: Commercial *S.cerevisiae*, S6: ATCC Mixture, S7: commercial mix	Water:pollen (1:1)	37°C, 72 h	Optimal: Choozit® (mixture of *St. thermophilus, L. delbrueckii* subsp. *lactis* and subsp. *bulgaricus*) and Commercial mix; ↑ total phenolics and flavonoids content and antioxidant activity ↑ LAB content which gave it a probiotic characteristic	(68)
Polyfloral BP	Fermented BP and BB	BP fermented with and without *L. rhamnosus*	Water:pollen (1:5)	32°C, 288 h for 12 days	↑ total phenolics and flavonoids content and antioxidant activity in BP fermented with *L. rhamnosus*	(97)
BP from Colombia	Fermented BP	*L. acidophilus, L. plantarum, S. cerevisiae*, and mixture of *L. plantarum* and *S. cerevisiae*	Fresh BP, dry BP: Water: pollen ratio 2:1 and 1:1	35 and 40°C for 72 h	BP: water (1:1) at 35°C ↑ bioactive compounds compared to raw material	(21)
pollen, borage honey	BB	Without selected strains	T0: BP without inoculum, T1: natural BB, T2: BP + 5% inoculum, T3: BP + 10% inoculum, T4: BP + 15% inoculum, T5: BP + 20% inoculum	35°C, 480 h	↑ acidity (4.83%) in T5 similar to natural BB	(69)
BP from Cuba	Fermented BP	*L. acidophilus, L. casei, Loigolactobacillus coryniformis, L. delbrueckii, L. plantarum*	pollen silage, honey	35°C, 360 h	*L. plantarum* and *L delbrueckii* at 36–40% humidity were the most promising, with > 1% lactic acid and total inhibition of *E. coli*.	(70)
BP from Colombia	Fermented BP	*L. acidophilus, L. paracasei subsp. paracasei*, and two mixed cultures: Yomixtm 205 Lyo And Choozit Tm My800 from Danisco® (108CFU/g)	pollen:water (2:1)	35°C, 72 h	*L. acidophilus* was the most promising inoculum; 121°C/15 min treatment improved the microbiological characteristics; ↑ acidification capacity (0.16 g lactic acid/kg*h) and survival rate (108 CFU/g 24 h in incubation) in *L. acidophilus*	(98)
BP and honey	Probiotic product	*Limosilactobacillus fermentum* BS2*, L. plantarum* BS1, BS3*, L. paracasei* BS6*, Bifidob. bifidum* BS4, BS5	B1: unground BP, honey, water (4:1:1); B2: ground BP, honey, water (4:1:1); B3 –unground pollen, honey, water (4:1:1) and 1% lactulose; B2: ground	37°C for 48 h and incubation for 7 and 14 days	Optimal method using ground pollen and inulin; ↑ probiotic viability in B2; ↑ antioxidant activity in B2 after 7 and 14 days; ↑ lactic acid content (%) after 4 days	(80)
			BP, honey, water (4:1:1) and 1% lactulose			
Canola BP	Breaking the pollen wall	*Ganoderma lucidum* and *Saccharomyces cerevisiae*	BB1: *G. lucidum* fermentation: pH = 5.5; BB2: *S. cerevisiae* fermentation: pH = 5.0	BB1: 30°C; 8 days; BB2: 36°C; 8 days	↓ reducing sugar level ↑ CMCase activity of G. lucidum at day 3 ↑protease activity in the 5^th^ day for *G. lucidum* and in the 6^th^ day for *S. cerevisiae* ↑ pectinase activity in BB1 (83.99 ± 2.81 U/mL) at day 4 and in BB2 (90.51 ± 5.53) U/mL at day 5 Optimal laccase activity in BB1 at day 3 (546.30 ± 8.18 U/L)	(99)
Honey, BP (pellets or ground)	Symbiotic product	Fermented product with *L. plantarum* and *L. acidophilus* administration daily in Wistar rats (*n* = 10): G1: 2 mg/kg; G2: 20 mg/kg; G3: 200 mg/kg; G4: control	BP1: 20 g unground BP, 3 g honey, 5 mL distilled water; BP2: 20 g ground BP, 3 honey, 5 mL distilled water; BP3: 20% unground BP, 3%honey; BP4: 20% ground pollen, 3% honey	37°C, 72 h	↑ lactic acid content in BP2 after 24, 48 and 72 h; ↑ viability of *L. plantarum* and *L. acidophilus* strains in BP2 and BP4; ↓ cholesterol levels at an intake of 20 mg kg−1 of BP; ↑ body weight after 4 weeks in the BP1 and BP2 groups	(75)

*CFU, colony forming units; FA, fatty acids; PUFA, polyunsaturated fatty acids; ↑, increase; ↓, decrease*.

The most important mechanism for the fermenting of microorganisms is carbon metabolism, in which carbohydrates are converted into essential compounds such as alcohols, acids, and carbon dioxide as the main end–products (100). To produce lactic acid, *L. bulgaricus*, and *Streptococcus thermophilus* consume sugar up to 3.2%, but in an acidic environment, those species become inactive (101). In this aspect, following fermentation, the levels of maltose and turanose decreased. The contents of free and total phenolic compounds increased by 18.3 and 17.8% after fermentation, whereas the content of bound phenolic compounds decreased. Fermentation with various microbial strains was demonstrated to enhance the quality of phenolic compounds in BB attributed to BP's structure (23, 102). Most bioactive compounds can be changed during fermentation due to the microbes' metabolic actions; thus phenolic acids are released under acidic conditions—field (103). Furthermore, peptides play critical roles in enhancing food taste and flavor. Most people cannot eat BP due to its distinctive aroma; however, active–taste peptides may solve this limitation (104).

BP is well-known to be rich in nicotinic, pantothenic acids, and riboflavin, while the riboflavin content of BP is the highest among all plant-based materials. The contents of riboflavin, nicotinamide, nicotinic acid, and amino acids were increased after fermentation in both pollen mediums, emphasizing the fermentation with *L. bulgaricus* and *S. thermophilus* can enhance vitamin content (105). In addition, the proteins degrade into smaller molecules through the fermentation process, making digestion and consumption much more accessible. Aside from that, wall-breaking pollen demonstrated more significant advantages in nutrient transformation during the fermentation process (96). In 2019, Di Cagno et al. (45) attempted to reproduce the natural process of BB fermentation by performing an actual solid-state fermentation process with BP as the primary substrate. Furthermore, they established a biotechnological protocol in which the role of LAB is precisely emphasized, and specific parameters, like temperature, pH, use of selected starters, time, influence the quality of the product developed at the fermentation end (Table 3).

Since fermentation is the most cost-effective method to improve the availability of high-quality nutrients for the human body, Knazovická et al. (73) simulated the function of bees with BP using a natural fermentation model analyzed the final product, which they dubbed pollen can. Physico-chemical analyzes of pollen can (BB) showed a 60% increase in water content, a 40% increase in free acidity, and a 17% decrease in pH, along with a reduction of 2% of fat compared to the results of analyzes performed on raw BP. The drop in pH is caused by lactic and acetic acid fermentation and alcoholic fermentation, thus ensuring a defensive role and spoilage avoidance of BB.

In 2019, another study found a potential improvement in the nutrient content of the resulting product through the solid fermentation of BP with LAB (96). The study aimed to obtain a nutritionally improved novel natural food product from BP that can be used as a nutritional supplement or a valuable ingredient in other foods. The LAB produced lactic acid by using the carbohydrates found in BP. During fermentation, the lactic acid content increased slightly after 168 h, with the final level of 6.10%, while in control, it remained unchanged, and the total sugar content decreased by 31.60%. The protein level in BP before fermentation was 26.80 mg/g, which increased by 12.53 mg/g after fermentation. Further details can be seen in Table 3.

### Yeast on the Biotechnological Process and Probiotic Usage

Foods have been fermented to enhance their organoleptic and nutritional characteristics from ancient times. In the 20^th^ century, industrial microbiology grew even further when new opportunities for producing a wide range of goods by fermentative processes emerged (90). Recent developments in yeast taxonomy, ecology, biochemistry, genetics, and molecular biology have piqued the interest in their role and importance in beverages and foods. This has led to a better understanding of the fermentation functions of well-known products and their role in the fermentation processes of other substrates (93). During their development in beverages and foods, they absorb nitrogen and carbon substrates and produce a wide range of volatile and non-volatile metabolites that influence the product's chemosensory properties. At the same time, other yeasts create extracellular amylases, proteases, lipases, and pectinases which also affect the aroma and texture of products (106). Yeast enzymatic activity is now critical in the processing of wide varieties of fermented food products; thus through a fermentation process under anaerobic or oxygen rich-conditions, it is likely to obtain ethanol and carbon dioxide (107). Compared to LAB, yeasts are not especially nutritionally demanding, but even so, the presence of simple compounds such as fermentable sugars, vitamins, minerals, amino acids, and oxygen stimulate their growth (108).

During a study, Yan et al. (96) fermented BP with various yeast or yeast mixtures the following fermentation. The overall phenolic compound contents of BP or wall-breaking pollen increased in the next order: fermentation with a microbial combination> fermentation with yeasts > LAB fermentation. The findings indicate that peptides with lower molecular weight significantly increased throughout fermentation. Peptides are either released during protein hydrolysis or formed by microorganisms involved in fermentation (97). A surprising aspect is that the following fermentation with yeasts, peptides with lower molecular weight (<1,000 Da), also known as oligopeptides, carry out a variety of active functions such as blood pressure regulators, anticoagulants, and antioxidants (109).

Yeast fermented BP may contain more carbohydrates, generate oligopeptides, free essential amino acids, PUFAs, and polyphenols than BP fermented by LAB; thus, yeast fermentation is a viable option for improving BP's nutritional properties (29). In this aspect, Zhang et al. (99) investigated the fermentation mechanisms of Canola BP by *S. cerevisiae* and *Ganoderma lucidum* to facilitate the breaking of the pollen wall. The pollen wall cannot fully decompose in the human digestive system, and its contents are only released through the germinal aperture, resulting in a nutrient consumption reduction. As a result, deterioration of the pollen wall is needed to maximize the use of nutrients present inside BP (58). On day 1, the Canola BP coating disappeared, the germinal apertures continued to expand with fermentation until the 8^th^ day when the contents were released, and the wall's structure was damaged. *G. lucidum* broth comprises higher amounts of nutrients compared to *S. cerevisiae*. The results showed a suitable fermentation method for breaking the pollen wall and releasing nutritional compounds (e.g., polysaccharides, ganoderic acid) (110).

*Hanseniaspora uvarum*, a unique yeast species that can grow at 1.5 pH (most yeasts grow better at pH 4.5–7.0), along with *A. kunkeei*, was used by Di Cagno et al. to reproduce the natural process of BB by performing an initial solid-state fermentation process with BP as the main substrate (45). Yeasts were detected at the uppermost cell densities in raw BP throughout their first 20 days of storage and unstarted BP throughout their last 20 days. *H. uvarum* pectin-degrading enzymes were required to break down the pollen walls, resulting in nutrient release; this hypothesis emphasizes the close interaction between LAB and other microbial groups during BB fermentation (111). This study linked the growth process of BB to the active role of native LAB in collaboration with yeasts. Furthermore, it highlighted the critical role of *A. kunkeei* and the development of a fermentation protocol for BP that nearly reproduces the natural process of BB fermentation, resulting in a well-constructed and consistent fermented product with a high nutritional value, ideal for human consumption (45). Further details can be seen in Table 3.

The present studies indicate the favorable influence of BP solid-state fermentation on the nutritional content of the resulting product, BB, by boosting the bioavailability and digestibility of nutrients and bioactive substances. Furthermore, since all of the studies listed above have achieved positive features in their field of research, biotechnological BB can be considered a different food with a great source of natural nutrients and a product that is beneficial to human nutrition and health (14, 112).

## Potential Therapeutic Properties of LABs Found in BB and/or Used in the Fermentation Process of BP

Probiotics from bee products possess several health benefits, including controlling gastrointestinal infections, improvement in lactose metabolism, anticarcinogenic and antimutagenic properties, cholesterol reduction, immune system stimulation, and improvement in inflammatory bowel disease (Table 4). Probiotics from BB produce bacteriocins and short-chain fatty acids, which help lower gut pH, enhance the available nutrients, colonize the colon with available microorganisms, stimulate mucosal barrier function, and increase immunity (Table 5). Moreover, several studies demonstrated the stimulatory effect of probiotics of the natural and gained immune response by inducing secretory and systemic IgA secretion (135–137).

**Table 4:** Therapeutic properties of probiotic bacterial isolates found in BB and/or used in the fermentation process of BP.

**Functional properties**	**Product**	**Isolated or used strains**	**Health effects**	**Reference**
Antibiotic susceptibility	*Apis mellifera* BB	*A. kunkeei* K18, K34 and K45, *Lact. rhamnnosus* GG (ATCC 53103)	K18, K34 resistant to Ampicillin and Kanamycin	(81)
	*Apis mellifera* BB	*Companilactobacillus musae* SGMT17, *Companilactobacillus crustorum* SGMT19, SGMT20, *Companilactobacillus mindensis* SGMT22	↑ resistance to Vancomycin, Teicoplanin, Kanamycin, Streptomycin, Gentamicin	(113)
	n.a.	LGG	↑ decrease in optical density of preformed biofilms of Corynebacterium and antibiotic-resistant gram-negative bacteria	(114)
	*Apis mellifera* BB	*A. kunkeei* AP-2, AP-8, AP-13, AP-15, AP-16, AP-18	↑ resistance to Kanamycin and Streptomycin	(115)
Antibacterial	Fresh *BB*	*Metschnikowia pulcherrima*	↑ activity against *Candida parapsilosis, Debaryomyces occidentalis, Proteus vulgaris*, and *S. cerevisiae*	(116, 117)
	*Apis mellifera* BB	LGG and isolated EPS	↑ inhibition against *C. albicans* ↑ inhibition against hyphal formation of *C. albicans* Isolated EPS reduced adhesion of *C. albicans* to VK2/E6E7 (30%) and Calu-3 (27%) at 200 μg/mL	(118)
	BB of stingless bee *H. itama*	*Bacillus safensis* BB2, *Bacillus amyloliquefaciens* BB5, *Bacillus pumilus* U1, *Bacillus cereus* U22, MPS3	↑ Haemolytic activity	(113)
	BB of stingless bee *H. itama*	*C. musae SGMT17*	↑ antibacterial activity against *Escherichia coli, Staphylococcus aureus, Salmonella typhimurium, Pseudomonas aeruginosa*	(113)
	BB of stingless bee *H. itama*	*Leuconostoc mesenteroides* U39	↑ antibacterial activity against *E. coli, S. aureus, Salm. typhimurium, Pseud. aeruginosa*	(113)
	BB of stingless bee *H. itama*	*C. crustorum* SGMT20	↑ antibacterial activity against *Pseud. aeruginosa*	(113)
	*Apis mellifera* BB	*Bacillus amyloliquefaciens SGMT3*	↑ antibacterial activity against *Staphylococcus aureus*	(113)
	*Apis mellifera* BB	*Leuconostoc mesenteroides* subsp. *suionicum* strain M6S3B6	↑ inhibitory activity against *Pseudomonas aeruginosa* (ATCC 27853) *Escherichia coli* (ATCC 11775) *Bacillus subtilis* (ATCC 21332) *Staphylococcus aureus* (ATCC 25923) *Klebsiella pneumoniae* (KU593478)	(113)
	BB *Hedera helix*	*L. plantarum* PLB1	↑ inhibition in *Bacillus megaterium* F6	(45)
	BB *Hedera helix*	*A. kunkeei* PFB13, PFA7, PFA35	↑inhibition in *Bacillus megaterium* F6 and *Pantoea agglomerans* DTB8 Moderate to ↓ inhibition in *Listeria monocytogenes* ATCC 19115, *Escherichia coli* DSM 30083, *Serratia marcescens* DR8 and DR10	(45)
	BB *Hedera helix*	*A. kunkeei* PLA14	↑ inhibition in *Bacillus megaterium* F6, *Pantoea agglomerans* DTB8, *Escherichia hermannii* PS2	(45)
	BB *Hedera helix*	*A. kunkeei* PLA21	↑ inhibition against *Bacillus megaterium* F6, *Pantoea agglomerans* DTB8 and *Serratia marcescens* DR10	(45)
	BB *Hedera helix*	*A. kunkeei* PFA15	↑ inhibition in *Bacillus megaterium* F6	(45)
	*Apis mellifera* BB	*A. kunkeei* AP-2, AP-8, AP-13, AP-15, AP-16, AP-18	↑ levels of antibacterial activity against *S*. *aureus* ATCC 29213, *B*. *cereus* ATCC 11778, *E*. *coli* ATCC 25922, and *S*. *typhimurium* RSSK 95091	(115)
Bile tolerance	BB of stingless bee *H. itama*	*Fructobacillus fructosus* U47, *C. mindensis* SGMT22	↓ bile tolerance (75.66% survival rate) after exposure to 0.3% bile for 4 h	(113)
	*Apis mellifera* BB	*A. kunkeei* AP-2, AP-8, AP-13, AP-15, AP-16, AP-18	↑ inhibition levels (60–80%)	(115)
pH survival rate	*Apis mellifera* BB	*A. kunkeei* AP-2, AP-8, AP-13, AP-15, AP-16, AP-18	↑ tolerance to low pH conditions	(115)
Bile tolerance	*Apis mellifera* BB	*Leuconostoc mesenteroides* U39, *F. fructosus* U45, *C. musae* SGMT17, *C. crustorum* SGMT19, SGMT20, *E. faecalis* MPS15	↑ bile tolerance after exposure to 0.3% bile for 4 h	(113)
Pepsin tolerance	*Apis mellifera* BB	*C. musae, C. mindensis, C. crustorum*	↑ survivability rate (98.20–100%) after 3-h exposure to pepsin	(113)
Pancreatin tolerance	*Apis mellifera* BB	*C. musae, C. crustorum*	↑ survival rate of L. musae SGMT 17 (99.38%) followed by L. crustorum SGMT20 (99.23%) to exposure to pancreatin	(113)
Cell autoaggregation	*Apis mellifera* BB	*C. mindensis* SGMT22	↑ autoaggregation ability (41.16%)	(113)
	*Apis mellifera* BB	*A. kunkeei* AP-2, AP-8, AP-13, AP-15, AP-16, AP-18	↑ autoaggregation ability (65%)	(115)
Cell surface hydrophobicity	*Apis mellifera* BB	*Lc. mesenteroides* U39, *C. mindensis* SGMT22 and *C. musae*	↑ cell surface hydrophobicity (80.52, 74.51, and 59.41%, respectively)	(113)
Antifungal	BB *Hedera helix*	*A. kunkeei* PFB13	↓ inhibition in *Aspergillus versicolor* CBS 117286	(45)
	BB *Hedera helix*	*A. kunkeei* PLA13	↑ inhibition in *Aspergillus versicolor* CBS 117286 and *Penicillium roqueforti* DPPMA1	(45)
	BB *Hedera helix*	*L. plantarum* PLB16	Moderate to ↓ inhibition in *Penicillium albocoremium* CBS 109582 and *Penicillium roqueforti* DPPMA1	(45)
	*Apis mellifera* BB	*A. kunkeei* AP-2, AP-8, AP-13, AP-15, AP-16, AP-18	↑ activity of all tested strains against *A. paraciticus* and *B. cinerea* ↑ activity of *A. kunkeei* AP-2 against *A. alternata, A. paraciticus* and *B. cinerea* ↑ activity of *A. kunkeei* AP-20 against *F. oxysporum, A. paraciticus* and *B. cinerea*	(115)
	n.a.	LGG	↑ activity against *Candida* hyphae formation	(118)
Safety and probiotic potential	*Camellia sinensis* BB	*Lactobacillus jensenii, F. fructosus and Lactococcus plantarum*	Production of organic acids in BB	(119)
	*n.a*.	LGG	↑ auto-aggregation of LGG after 24 h incubation ↑ biofilm formation in TSB with 0–1% glucose ↑ Co-aggregation percentages with *E. coli* DSM 5698, *E. coli* K12-DH5, *P. mirabilis* ATCC 29906, *Ec. faecalis* ATCC 2912, *S. aureus* ATCC 29213, and *Lb. acidophilus* ATCC 4356 evaluated after 5 and 24 h of incubation	(120)
Caries prevention	*n.a*.	LGG	↑ viable cell numbers with glucose and sucrose in 64.5-h multispecies experimental oral biofilms ↓ pH values of spent media at each time point with lactose No harmful effects detected on dental hard tissues	(121)

*NCM460, normal mucosa intestinal cells; IL, Interleukin; APEC, Avian pathogenic E. coli; HGC-27, Gastric carcinoma cells; ODC, Ornithine decarboxylase; SSAT, spermidine/spermine N1-acetyltransferase; AGS, gastric adenocarcinoma cells; LGG, Lacticaseibacillus rhamnosus GG (ATCC 53103); HCT-116, human colon carcinoma cell line; ↑, increase; ↓, decrease*.

**Table 5:** *In vitro* studies regarding the potential therapeutic properties of LABs found in BB and/or used in the fermentation process of BP.

**Disorder**	**LAB strain**	**Study model**	**Effects**	**References**
Bladder cancer	Live and Lyo LGG (lyophilized)	MB49-PSA cells	↑ TNF-a in live LGG (414.27 ± 251.96 pg /mL) and Lyo LGG (318.46 ± 208.28 pg /mL) ↑ IL-12p40 in live LGG (76.45 ± 2.97 pg /mL) and Lyo LGG (102.30 ± 31.64 pg /mL) ↑ IL-10 in live LGG (193.14 ± 93.35 pg /mL) and Lyo LGG (393.42 ± 225.19 pg /mL) ↑ number of dendritic cells to the bladder ↓ number of dendritic cells in local lymph nodes ↑ immune cell recruitment into the bladder	(122)
Gastric adenocarcinoma	*LGG*	HGC-27 cells	↓ODC mRNA and activity, polyamine content, neoplastic proliferation after 24 and 48 h ↑ SSAT mRNA and activity	(123)
	Live and heat-killed *LGG*	AGS cells	↓reduced adhesion of *H. pylori* by 50% on AGS cells at a concentration of 10^10^ CFU/mL ↓ IL-8 (5,500 ± 1,600 pg /mL) with live LGG	(124)
	Viable and heat-killed LGG (10^8^ CFU/mL)	HGC-27 Gastric cell line	↑ adhesion of LGG (75.4%−90.9%) ↑ proliferation activity after 48 h ↑ proapoptotic effect of viable and heat-killed LGG after 24 and 48 h	(125)
	LGG homogenate (1 × 10^8^, 5 × 10^7^ and 2 × 10^7^ CFU/mL)	HGC-27 Gastric cell line	↑ antiproliferative action with increasing concentrations of LGG after 24 h and 48 h of treatment ↓ conversion of the MTT tetrazolium salt at 1 × 10^8^ CFU/mL compared with the untreated control cells after 24 h ↓ in [3H]-thymidine incorporation in DNA of cells with 5 × 10^7^ CFU/mL compared with the untreated control cells ↓ spermine, spermidine and total polyamine content 1 × 10^8^ CFU/mL compared to untreated control cells after 24 and 48 h ↑ Bax/Bcl-2 ratio compared to untreated cells with 1 × 10^8^ CFU/mL after 24 h and 48 h	(126)
Colorectal cancer	Live and UV-Inactivated LGG	Caco-2 Cells	↓ IL-8 expression by both LGG ↓ Ub-IκB expression by UV-inactivated LGG	(127)
	LGG	HCT-116	↓ cell invasion to 49% ↓ MMP-9 activity to 72% ↑ ZO-1 protein levels to 170% ↓ cell invasion to 30%	(128)
	*L. plantarum*	NCM460 cells	↓ IL-17F (0.17–1.00 pg/mL) and ↓ IL-23 (16.9–18.6 pg/mL) in inflamed NCM460 cells	(83, 129)
	Viable and heat-killed LGG (10^8^ CFU/mL)	DLD-1 Colon Cell Line	↑ adhesion of LGG (90%−98%) ↑ proapoptotic effect of viable and heat-killed LGG after 24 and 48 h	(125)
	Live (1 × 10^6^ CFU/mL) and heat-killed (1 × 10^8^, 1 × 10^9^ and 1 × 10^10^ CFU/mL) LGG	Caco-2 cells	↓ chemoxine (CCL20, CXCL8 and CXCL10) expression in Caco-2 cells ↑ suppression of *E. coli*-induced expression of all 3 chemokines ↑ suppression on the expression of CXCL8 in Caco-2 cells after stimulation with PGN ↑ suppression on the expression of CCL20 and CXCL10 in Caco-2 cells after stimulation with PGN and flagellin ↑ HSPA1 and HSPA6 expression in Caco-2 cells with (10 ^10^ CFU/mL) of heat-killed LGG	(130)
	Freeze-dried LGG	Human T84 colon epithelial cells	↑ expression of COX2 protein in a concentration-dependent manner in T84 after 72 h	(131)
	Live and heat-killed LGG	Caco-2 cells	↓TER levels in Caco-2 cells ↑ TER levels (24 h post TNF-a stimulation) in the presence of LGG (10^5^) ↓ TER levels (24 h post TNF-a stimulation) in the presence of heat-killed LGG and LGG+ chloramphenicol (20 μg mL^−1^)	(132)
			↓ CXCL-8 and CCL-11 secretion from cytokine-stimulated epithelial monolayers	
Chronic colitis	LGG	RAW 264.7 macrophages and spontaneous mutant, RAW 264.7 gamma NO(–)	↑ inhibition of TNF-α production by LPS-activated macrophages ↑ inhibition of TNF-a production by LTA-activated macrophages	(133)
Irritable bowel syndrome	LGG	HT-29, Caco-2 cells, and intestinal epithelial cells	↑ SERT mRNA levels in HT-29 cells treated with increased concentration of LGG for 12 and 24 h ↑ SERT protein expression in HT-29 cells treated with increased concentration of LGG for 12 and 24 h ↑ SERT mRNA expression and protein levels of SERT in Caco-2 cells treated with increased concentration of LGG for 12 and 24 h ↑ SERT mRNA levels in mice intestinal epithelial cells at the 1st week ↓ SERT mRNA levels in mice intestinal epithelial cells at the 2nd week	(134)

*HCT-116, human colon carcinoma cell line; HGC-27, Human gastric cancer cell line; DLD-1, colon cancer cell line; Caco-2, human colon adenocarcinoma cell line; PGN, peptidoglycan; RAW 264.7, mouse monocyte/macrophage cell lines; LPS, lypopolysacharides; LTA, lipoteichoic acid; CXCL-8, interleukin-8; CCL-11, eotaxin; SERT, serotonin transporter*.

Multiple human and animal studies have been conducted and suggest that probiotics are GRAS and practical for clinical application on human diseases, such as acute pediatric diarrhea (138), rotavirus-related diarrhea (139), infantile colic (140), necrotizing enterocolitis in shallow birth weight infants (141), type 1 diabetes (142), allergic asthma (143) inflammatory bowel disease (144), bone loss (145), and bacterial vaginosis (146), and virus infection (147). Further details can be seen in Table 6.

**Table 6:** *In vivo* studies regarding the potential therapeutic properties of LABs found in BB and/or used in the fermentation process of BP.

**Functional properties**	**LAB strain**	**Treatment scheme**	**Treatment duration**	**Health effects**	**References**
Anti-inflammatory	*A. kunkeei* YB38	11 subjects took 1 g heat-killed YB38 once a day	4 weeks	Significant ↑ in SIgA concentration ↑ IgA production in mouse Peyer's Patch cells ↓ mitogenic activity and ↓ effect on IL-2 production in mouse spleen cells	(135)
	*A. kunkeei* YB38	*IFV sublethal infection in mice*: 6-week-old BALB/c mice divided into 2 groups (*n* = 100) administered daily oral doses of YB38 saline dilution (0 or 100 mg/kg) for 22 days *IFV lethal infection in mice*: 6-week-old BALB/c mice were divided in 3 groups (*n* = 60) and daily administered oral doses of 0, 10, or 100 mg/ kg of YB38 saline dilution	n.a.	↓ numbers of T cells, NK cells, neutrophils, and macrophages in the mice inoculated with the heat-killed YB38 treatment compared to control at 4, 4–8, 4–6, and 8 days after infection; IL-6 production was significantly suppressed in the heat-killed YB38-treated group ↑ IgA concentration in YB38-treated group ↓ tissue damage in YB38-treated group	(137)
	*A. kunkeei* YB38	29 female subjects consumed 0 (placebo), 2, 10, and 50 mg of heat-killed YB38	n.a.	↓ in intestinal levels of *Bacteroides fragilis*	(136)
	Capsule with LGG (10^9^)	home residents (*n* = 196; >65 years old) G1 (treatment, *n* = 100) = 2 capsules/day G2 (control, *n* = 96): placebo (calcium carbonate capsule)	6 months	↓ respiratory viral infections observed in 14 (15.0%) residents in G1 Moderate respiratory viral infections observed in 21 (22.9%) residents in G2	(148)
	LGG-derived protein (p40)	4–6 week-old C57BL/6 mice G1: gavaged with pectin/zein beads with p40 from LGG (10 mg per day) G2: pectin/zein beads	3 weeks	↑IgA level in LGG treatment group ↑*April* gene expression in MSIE cells	(128)
	LGG	Human intestinal mucus isolated from 114 fecal samples collected from healthy infants and adults	n.a.	↑adhesion properties to adult human mucus ↓ adhesion properties to neonatal and infant mucus	(149)
	LGG	20 ulceritive-colitis (UC) patients and 22 normal subjects) G1 (*n* = 10): negative control G2 (*n* = 12): dose of 1.2 × 10^10^ CFU/d LGG (2 packets/day) G3 (UC, *n* = 10): 1.2 × 10^10^ CFU/d) dose of LGG G4 (UC, *n* = 10): 2.4 × 10^10^ CFU/d dose of LGG	7 days	↑ adhesion in the normal colon after 7 days of LGG administration in G4 (6.83 ± 2.97) compared to control ↓ mucosal TNFα (1.68 ± 0.22) and IL-17 (1.05 ± 0.11) expression in G4	(150)
Safety and tolerability	LGG	15 volunteers (aged 66–80 years) received 2 capsules (10^10^) daily for 28 days and were followed through day 56	56 days	↓ adverse effects, most common were gastrointestinal (bloating, gas, and nausea) ↓ IL-8 during LGG consumption ↓ hemoglobin in 2 patients at day 28 ↑ Blood Urea Nitrogen in 2 patients at day 28 and 1 patient at day 56 ↑ White Blood Count in 1 patient at day 56 ↓ glucose level in 1 patient at day 28 ↑ glucose level in 1 patient at day 28	(151)
Immunomodulation	LGG	Female (4–6 weeks old) C57BL/6 mice (*n* = 12/group) immunized by oral gavage with 10^8^ CFU LGG on days 0, 14 and 28 G1: live LGG-GFP G2: LGG IL-2-GFP G3: wild type LGG G4: sterile PBS	28 days	↑ survival of LGG-IL-2-GFP ↑ cytokine gene expression at 12 h and ↓ by 24 h ↑ IgA producing B cells and CD86 positive DC in G2 ↑ GFP specific IgG titres in G2 ↑ GFP specific sIgA in fecal extracts from 24^th^ to 35^th^ day ↑ GFP specific CD8+ T cells in G2 ↓ GFP specific CD8+ T cells in G1 ↑ CTL activity in G1 and G2	(152)
Immnunotherapy against bladder cancer	LGG	C57BL/ 6 female mice aged 4–6 weeks divided in 5 groups (*n* = 50) G1: live LGG G2: Lyo LGG G3: oral 1 × 10^8^ live LGG 1 day before intravesical Lyo (O + I) LGG G4(control): intravesical instillations of PBS G5 (control): oral PBS 1 day before intravesical PBS	6 weeks	↑ PF4, XCL1, and P-selectin in LGG-treatment groups ↓ OPN, Pro-MMP9, Thymus CK-1, and VEGFR2 in LGG-treatment groups ↑ bladder VEGF-D ↑ splenic Mac3+ cells in LGG-treatment groups ↑ number of cured mice	(122)
Intestinal crypt loss	LGG	8-week-old C57BL/6 by gavage of Gavage of 5 × 10^7^ LGG	n.a.	↓ epithelial apoptosis ↓ radiation-induced epithelial injury ↑ crypt survival	(153)
Immunomodulation	LGG (ATCC 53103)	Forty-five 6-week-old female BALB/c mice divided in 3 groups G1 (control): intragastrically administered PBS (200 μL) every other day for 7 total treatments G2 (LLGG): intragastrically administered LGG every other day for 7 total treatments (doses from 10^3^ to 10^9^CFU) G3: intragastrically administered 10^9^CFU every other day for 7 total treatments	n.a.	↑ OTU, Chao1, ACE, and Shannon indices ↓ Simpson index ↑ abundances of intestinal *Firmicutes, Bacteroides* and *Actinomycetes* ↓ intestinal *Proteobacteria* ↑ immunity ↑ expression of Th1-type cytokines (IFN-γ) and Th2 cytokines (IL-4) in CD4^+^ T cells ↑ expression of IL-17 in CD4^+^ T cells ↑ percentage of CD4^+^CD25^+^Foxp3^+^ Treg cells	(154)
Hepatotoxicity	LGG (ATCC 53103)	Twenty-four male Holstein calves divided in 3 groups received oral administration in 50 mL of phosphate-buffered saline G1: untreated control G2: 4.80 mg of AFB_1_ G3: 1 × 10^10^ cfu of LGG suspension and 4.80 mg of AFB_1_ Treatment duration: 14 days	14 days	↑ ADG in G3 nad G1 compared to G2 ↓ AST and LDH compared to G2 ↓ concentration of AFB_1_ and AFM_1_ in rumen fluid, urine and plasma in G3 ↑ concentration of AFB_1_ and AFM_1_ in feces	(155)
Diarrhea treatment	LGG	Four weeks old Female Sprague Dawley rats (n = 64) divided in 4 groups G1: control G2: oral daily dose of LGG 1 × 10^9^ CFU lactobacilli/1 ml	25 weeks	↓β-catenin expression in G2 and G4 ↓ NFκB-p65, COX-2 and TNFα inflammatory proteins ↑ pro-apoptotic proteins Bax casp3 and p53 ↓ VEGFα expression in G4 ↓ tumor incidence in G2	(156)
		G3: weekly single dose of 40 mg/kg body weight of DMH intraperitoneally for 10 consecutive weeks G4: weekly single dose of 40 mg/kg body weight of DMH intraperitoneally for 10 weeks and daily oral dose of LGG 1 × 10^9^ CFU lactobacilli/1 ml for 25 weeks			
Atopic dermatitis (AD)	LGG	39 infants with AD divided in 2 groups G1 (*n* = 19): 5.01 × 10^7^ CFU/g LGG to achieve a daily intake of 3.4 × 10^9^CFU G2 (control): Casein hydrolysate	3 months	↓ number of Ig-secreting cells in G1 ↓ IgA- and IgM-secreting cells in G1 from 1–3 months ↑ IgA- and IgM-secreting cells in G2 ↑ Number of Bifidobacterium species in G1 compared to control ↑ % colonization with *Bifidobacterium longum* in G1 compared to control ↓ % colonization with *B. fragilis* compared to control	(157)
	LGG	105 pregnant women with AD divided in 2 groups: G1 (*n* = 50): 5 × 10^9^ CFU/g LGG twice daily G2 (*n* = 44):placebo	4–6 weeks before delivery and a postnatal period of 6 months	risk of AD in children on probiotics relative to placebo was 0.96 no difference between groups regarding the cumulative incidence of atopic symptoms no difference in total IgE concentrations or numbers of specific sensitization to inhalant allergens between groups	(158)
	Freeze-dried LGG	Female NC/Nga mice divided in 2 groups G1 (*n* = 17): control G2 (*n* = 16): LGG 4 x 10^9^ CFU/g (~30–50 mg/adult mouse)	12 day of pregnancy to 12 weeks after birth	↓ grades of dermatitis G2 compared to control ↑ suppression of onset and development of atopic leasions in G2 compared to control ↓ mast cells number and eosinophils in G2 compared to control ↑ IL-10 levels compared to control ↑ expressions of IFN-g and IL-4 compared to control	(159)
	LGG	G1 (*n* = 27): Cow's milk elimination diet + formula milk with or without LGG G2 (*n* = 11): LGG (5 × 10^8^ CFU/g or 2 × 10^10^ CFU twice/day) during breastfeeding	4 weeks	↓SCORAD in probiotic group No significant difference between groups after 2 months	(160)
	LGG	G1 (*n* = 52): probiotic (1x10^10^ CFU/g LGG + 0.01 mg of vitamin D3 + 0.6 mg zinc) G2 (*n*= 44): placebo	n.a.	↓ reduction in SCORAD in probiotic group	(161)
Alcoholic liver disease	LGG supernatant	10-weeks old male C57BL/6 mice G1: alcohol (5% w/v, AF, *n* = 6) G2: isocaloric maltodextrin (PF, *n* = 6) G3: alcohol + 10^9^ CFU/ml LGG (AF + LGG-s, *n* = 6)	5 days	↓ Serum ALT and AST levels in G3 compared to G1 and G2 ↓ claudin-1 and ZO-1 mRNA expression in G3 compared to control and G2 ↑ villus-crypt junction in ileum ↓*E. coli* protein level compared compared to alcohol exposure in G2 ↓ frequency of T_H_17 in G3 compared to G2 ↓ serum IL-17 level in G3 compared to G2	(162)
	LGG supernatant	9-week old Male C57BL/6N mice G1: Control G2: alcohol at 6 g/kg body wt via gavage G3: 1 × 10^9^ CFU/mL + alcohol	5 days	↓ Hepatic tissue TG levels after 6 h alcohol exposure in G3 compared to G2 ↓ plasma ALT and plasma LDH in G3 compared to G2 after 1.5 and 6 h ↓ liver and ileum reactive oxygen species (ROS) formation compared to G2 ↓ plasma endotoxemia, ileum permeability in G3 compared to G2 ↑ mRNA levels of ileum tight junction proteins and adaptors and of ileum mucus protecting factors in G3 compared to G2 ↑ hypoxiainducible factor (HIF)-1α/2α expression in G3	(163)
	LGG supernatant	Male C57BL/6 N mice divided in 3 groups G1 (PF): isocaloric maltose–dextrin G2: alcohol-fed G3: alcohol + LGG (10^9^ CFU/mouse/day)	8 weeks	↓ MPO activity, TNFα protein levels and TNFα mRNA expression in G3 and G1 compared to G2 ↓ Cyp2E1 mRNA and protein levels in G3 and G1 compared to G2 ↓ hepatic TLR (toll-like receptors) mRNA expression	(164)
Alcohol-induced liver injury	LGG supernatant	Male C57BL/6N mice divided in 3 groups G1 (PF): isocaloric maltose–dextrin G2: alcohol-fed G3: alcohol + LGG (10^9^ CFU/mouse/day)	8 weeks	↑bw, ITF and VEGF ↑ mRNA levels of ZO-1, claudin-1, and occluding ↓ Epithelial Cell Permeability in Caco-2 Cells ↓ Alcohol-Induced ROS Formation in the Ileum and in Caco-2 cells	(165)
Non-alcoholic fatty liver disease (NAFLD)	LGG	6 weeks old female C57BL/6 mice (*n* = 24) divided in 4 groups G1: water and mouse breeding (MZ)-diet G2: 30% fructose solution+ enriched MZ-diet G3: LGG (5.2*107 CFU/g bw daily in water and MZ-diet G4: 30% fructose solution with LGG in water and enriched MZ-diet	n.a.	↓ ALT in G3 compared to G2 ↓ liver fat accumulation in G3 and G4 ↓ Hepatic ChREBP, ACC1 and FAS mRNA expressions in G3 and G4 compared to high-fructose diet (G2) ↓ TNF-α, IL-1β (B) and IL-8R (C) mRNA expressions ↑ occludin, and claudin-1 molecules ↓ pIkB kinase protein expression and LPS	(166)
Alcohol-induced intestinal oxidative stress and liver injury	Live LGG	Male Sprague–Dawley rats G1 (*n* = 11): alcohol gavage (~2–3 mL) twice daily (initial dose: 2 g/kg/day; final dose: 8 g/kg/day)+ powdered rat chow G2 (*n* = 9): alcohol gavage+ live LGG (2.5 × 10^7^/once daily) G3 (control, *n* = 5): isocaloric amount of dextrose, by gavage G4 (*n* = 3): dextrose + intragastric feedings of live LGG	10 weeks	↓ liver necroinflammation score (%) in G2 and G4 compared to G1 ↓ liver MPO levels and liver fat content in G2 and G4 compared to G1 ↓ liver carbonyl and nitrotyrosine levels in G2 and G4 compared to G1 ↓ intestinal permeability to sucralose in G2 and G4 compared to G1	(167)
Gastroenteritis	LGG	Children aged 6 months to 5 years positive for either rotavirus (G1 and G2, *n* = 82)or *Cryptosporidium* species (G3 and G4, *n* = 42) G1: probiotic:1 × 10^10^CFU/capsule LGG+ 170 mg of microcrystalline cellulose G2: 170 mg cellulose G3: LGG+ cellulose G4: 170 mg cellulose	4 weeks	↓ repeated diarrheal episodes in G1 (25%) compared to G2 (46%) ↓ impaired intestinal function in G1 (48%) compared to G2 (72%) ↑ IgG levels postintervention in G1 ↑ improvement in intestinal permeability in G3	(140)
Necrotizing enterocolitis	LGG	640 infants aged between 26.3 and 30.6 weeks	n.a.	↓ severe necrotizing enterocolitis and mortality in LGG-supplemented infants compared with non-supplemented infants	(168)
Bone loss	LGG	C57BL6/J mice divided in: G1 (control): normal saline (NS) vehicle by oral gavage G2: LGG+TDF (0.86 mg) daily G3: 5 × 10^8^ CFU LGG (10^9^ CFU/ml, dissolved in NS) twice a week G4: *E. coli*+TDF daily G5: TDF daily G6: ZOL+TDF daily	n.a.	↑ trabecular bone microarchitecture, cortical bone volume, and biomechanical properties in G2 compared to G5 ↑ intestinal barrier integrity, expanded regulatory T cells in G3 ↓ Th17 cells and osteoclastogenesis-related cytokines in the bone marrow, spleen, and gut ↑ expression of lysophosphatidylcholines	(169)
Gingival health	LGG	108 schoolboys (13–15 years) G1 (*n* = 54): placebo G2 (*n* = 54): 1 g probiotic mixture (LGG 4.4 × 10^8^ and BB-12 4.8 × 10^8^)	4 weeks	↓ gingival and plague index in G2 ↓ *Aggregatibacter actinomycetemcomitans* levels in saliva and plague ↓ total bacterial count in the saliva and plaque sample in both groups	(170)
Periodontitis	LGG	6–8 week old BALB/c female mice (*n* = 36) G1: Control G2: PD (periodontitis) G3: LGG (200 μl of 2-9 × 10^9^ CFU/ml of LGG) gavage + PD G4: LGG gavage G5: LGG oral + PD G6: LGG oral	44 days	↓mean bone loss (*p* > 0.05) in the LGG groups compared to G2 ↓ inflammatory scores in LGG groups compared to G2 ↓ TRAP positive cells (3.99 ± 2.05) and circulating levels of LIX in blood serum (61% lower) compared to G2	(145)
Human rotavirus (HRV) infection	LGG	Human gut microbiota (HGM) transplanted gnotobiotic (Gn) pig model G1: control with G2: 14 doses of LGG G3: AttHRV alone, G4: AttHRV + 9 doses of LGG G5: AttHRV + 14 doses of LGG	n.a.	↑ fecal and intestinal LGG counts in HGM transplanted Gn pigs after 23 days in G5 ↑ Rotavirus-specific IFN-γ producing T cell responses in ileum, IEL, spleen and blood ↑ Rotavirus-specific serum IgA and IgG antibody responses in LGG treated groups ↑ rotavirus-specific IgA antibody responses in small intestine contents of Gn pigs fed with higher LGG doses	(170)

*GFP, green fluorescence protein; Il-2, Interleukin-2; IgG, Immunoglobulin G; sIgA, secretory immunoglobulin A; CTL, cytotoxic T lymphocyte; CD8+ T cells, killer T cells; VK2/E6E7 ATCC-CRL-2616; normal human vagina epithelial cells; DMH, Dimethyl hydrazine; VEGFα, vascular endothelial growth factor A; NF-κB, nuclear factor kappa-light-chain-enhancer of activated B cells; COX 2, cyclooxygenase 2; TNFα, Tumor Necrosis Factor α; casp3, caspase3; p53, Tumor protein; PBS, Phosphate-buffered saline; PF, pair-fed; IgA, Immunoglobulin A; IgM, Immunoglobulin M; MSIE, mouse small intestine epithelial cells; APRIL, proliferation-inducing ligand; AFB_1_, aflatoxin B_1_; AFM_1_, aflatoxin M_1_; ADG,average daily gain; AST, aspartate aminotransferase; LDH, lactate dehydrogenase; ALT, Alanine aminotransferase; ITF, Intestinal trefoil factor; VEGF, vascular endothelial growth factor; BW, body weight; NAFLD, non-alcoholic fatty liver disease; ChREBP, carbohydrate response element-binding protein; ACC1, acetyl-CoA carboxylase 1; FAS, fatty acid synthase; IL-1b, interleukin 1 beta; IL-8R, interleukin 8 receptor; MZ-diet, mouse breeding diet; MPO, myeloperoxidase; HGM, human gut microbiota; Gn, transplanted gnotobiotic pig model; T1D, Type 1 diabetes; IgE, immunoglobulin E; SCORAD, Severity Scoring for Atopic Dermatitis; ZOL, zoledronic acid*.

So far, *Lacticaseibacillus rhamnosus* ATCC 53103 (formerly *Lactobacillus rhamnosus* LGG) is the most characterized, and studied probiotics, extensively used in the fermentation process of BP with promising results (171). It has multiple health benefits; for instance, it produces a biofilm that acts as a mucosa-protecting agent, enhances the intestinal pit survival through diverse soluble factors beneficial to the gut, diminishes apoptosis of the intestinal epithelium, preserves cytoskeletal strength along with pathogen inhibition, promotes immune awareness by reducing expression of inflammation markers and increasing production of IL-10 (interleukin-10), IL-12 and TNF- α (α-tumor necrosis factor) (172). Thus, the combination of BP fermented with different lactobacilli strains is significant for developing a novel product used both as food and medicine.

### Antibiotic Susceptibility

Several reports demonstrated that *Lactobacilli* strains have antibiotic resistance genes which can be transferred to the host gut of other bacteria. Therefore, it is significant to evaluate the antibiotic resistance properties of probiotics. Furthermore, characteristics linked to safety, survival in the host gut, and colonizing abilities are substantial to assess the proposed probiotic bacteria.

The prevalence of antibiotic-associated diarrhea varies based on diagnosis, with values between 5 and 30% in children and up to 70% in adults. In this aspect, treatment with LGG reduced the risk of antibiotic-associated diarrhea from 22 to 12% in adults and from 23 to 9.6% in children. In the LGG-groups, there was no need for antibiotic treatment discontinuation or intravenous rehydration (173). Korpela et al. evaluated whether long-term consumption of probiotics influences the antibiotic-associated changes in children's microbiota. Their results showed that long-term LGG-supplementation increased the levels of *Lactococcus, Prevotella*, and *Ruminococcus* and decreased the levels of *Escherichia*, as well as reduced the frequency of gastrointestinal complaints after the use of macrolides (174).

Salmonellosis is usually treated with antibiotics, which can cause side effects, including antibiotic resistance and enteric dysbacteriosis. Also, antibiotic therapy can worsen *Salmonella*-induced diarrhea. In this aspect, *Lactobacilli* strains are a promising alternative for Salmonella infection as they help prevent it, have fewer side effects, and have higher safety. Several *Lactobacilli* strains were found to attenuate the intestinal epithelial barrier dysfunction induced by *Salmonella* lipopolysaccharide (175). De Keersmaecker et al. demonstrated the potential antibacterial activity of LGG-spent culture supernatant (SCS) against *S. typhimurium* (176). Their results showed that several compounds, such as acetic, pyroglutamic, formic, and lactic acids, might be responsible for the antibacterial efficiency against *Salmonella* spp. Furthermore, infection with *Salmonella* spp. causes gut inflammation, which changes in the cytokine profile can detect. LGG reduced the levels of TNF-α in the colon while maintaining IL-10 and significantly reducing MPO (myeloperoxidase) levels (177).

### Antibacterial Activity

Several reports showed the antibacterial activity of BB due to its high content of polyphenols and LAB. Also, the antimicrobial potential of BB for the prevention and treatment of bacterial and fungal infections in animals and humans has been demonstrated in (1, 178). In a recent study, several polish BB samples exerted higher inhibitory potential against *Staphylococcus aureus* ATCC 25923 and ATCC 29213. Furthermore, several pieces effectively inhibited the growth of *S. aureus and* MRSA (methicillin-resistant *S. aureus*), strains at concentrations between 2.5 and 5.0% (179). In the following study, the bacterial isolates of mature BB exhibited moderate antagonistic effects against *S. aureus* ATCC 25923, ATCC 29213, *S. epidermidis* ATCC 12228, *P. aeruginosa* ATCC 27853, and lower results against *Escherichia coli* ATCC 25922 (180).

Despite the progress in antimicrobial therapy, sepsis and meningitis caused by *E. coli* K1 remain the primary cause of mortality and severe neurological morbidity, particularly in preterm and very-low-birth-weight infants (181). In this aspect, He et al. in 2017 evaluated whether LGG supernatant has a preventive effect against gut-derived systemic neonatal *E. coli* K1 infection in human colonic carcinoma cell line Caco-2 and neonatal rat models. The *in vitro* results showed that LGG up-regulated mucin and maintained intestinal integrity by blocking the adhesion of *E. coli* K1 to Caco-2 monolayer. In contrast, the *in vivo* results showed a decrease of *E. coli* K1 infection susceptibility in neonatal rats who received oral administration of LGG and reduced bacterial intestinal colonization (182). Furthermore, the LGG-treated rats had increased intestinal expression of MUC2 (intestinal mucin), Ki67 (intestinal epithelial cell proliferation marker), IgA (immunoglobulin A), ZO-1 (zonula occludens-1), and mucin layer, and decreased barrier permeability compared to untreated rats.

### Antiviral Activity

Influenza is an infectious disease that affects both animals and humans. The most common symptoms in humans are fever, coughing, muscle pain, chills, headaches, and weakness. In severe cases, it can cause pneumonia which can be fatal in children and the elderly. To investigate whether probiotics protect the host against influenza virus (IFV), BALB/c mice were infected with IFV A/PR/8/34 (H1N1) and administered intranasal LGG. It was shown that mice treated with LGG had lower symptoms frequency and higher survival rates than control. Also, increased mRNA expression of IL-8, TNF, and MCP-1 (monocyte chemotactic protein) were noticed in Ispirli and Dertli (183).

It was reported to reduce IL-2, IL-6, and IL-8, either by oral feeding or intraperitoneal injection with *Apilactobacillus* spp. The administration of heat-killed *A. kunkeei* YB38 reduced the symptoms of murine influenza pneumonia by enhancing SIgA production in mice infected with the influenza virus. The intake of *A. kunkeei* YB38 by healthy adults significantly improved secretory immunoglobulin A (SIgA) levels in saliva compared with baseline concentrations. Also, it decreased levels of the *Bacteroides fragilis* group.

### Gastrointestinal Disorders

Based on their viability, probiotics can survive in simulated gastrointestinal conditions. LAB and their bioactive specific cellular components exert many pivotal effects on the ecosystem of the human GI tract, including maintenance of the gut microbiota and control of the enteric mucosal pathogens, and modulation of the cell-mediated immune response. Auto-aggregation and cell surface hydrophobicity properties of the bacterial cells are essential characteristics to reveal their potential as probiotics which can be associated with the cells adhesion to the gastrointestinal, demonstrating their health-promoting functions. The EPS (exopolysaccharide) production levels in *A. kunkeei* strains from BB had values between 0.17 (*A. kunkeei* AP-13) and 2.79 (*A. kunkeei* AP-15) g/L (115). Also, supplementation with LGG in colicky breastfed infants resulted in reduced daily crying and fecal calprotectin and increased total bacteria and *Lactobacilli* compared to the placebo group (140).

LGG has beneficial effects on the organism as to be considered probiotic. In particular, LGG can withstand gastric acidity and bile salts, effectively adhering to the gastrointestinal mucosa. The ability to resist gastric acidity and bile salts is a consequence of the power of the bacterium to produce anti-stress proteins that give it greater survival capacity in intestinal transit after oral intake (Table 4).

As neonatal necrotizing enterocolitis (NEC) is one of the significant causes of mortality in premature infants, oral administration of *Bifidobacterium bifidum* has been suggested as a promising preventive treatment. Thus, *B. bifidum* (5 × 10^6^ CFU/day) administered to premature rat models of NEC decreased the ailment incidence from 57 to 17% and increased the levels of IL-6, mucin-3, and Tff3 (trefoil factor 3). The protective effect of *B. bififdum* is related to a decreased inflammatory reaction in the ileum, regulation of main components of the mucus layer, and intestinal integrity improvement (184).

### Bone Loss Prevention

Osteoporosis is a chronic syndrome of excessive skeletal fragility characterized by bone mass loss and bone micro-architecture deterioration. Patients with human immunodeficiency virus (HIV) (185) or subjected to antiretroviral therapy with tenofovir disoproxil fumarate (TDF) (186) are strongly correlated to bone loss. Recently, it has been suggested that the gut microbiota is linked to bone homeostasis (187). Thus, Liu et al. evaluated the effectiveness of LGG in attenuation of TDF-induced bone loss in 6-week old C57BL6/J mice. LGG treatment reconstructed the gut microbiota structure and increased the intestinal barrier integrity, proving an effective treatment against TDF-induced osteoporosis (169).

Periodontitis, a chronic inflammatory condition, affects the soft and hard tissues that support teeth. It is influenced by specific pathogenic bacteria (i.e., *Porphyromonas gingivalis* and *Fusobacterium nucleatum*), which have been shown to aggravate inflammation and alveolar bone loss in mice (188). In a recent study, LGG administration to *P. gingivalis* and *F. nucleatum*-inoculated mice reduced tissue inflammation in the duodenum and IL-6 levels in ileum compared to control. Oral gavage with LGG induced a protective effect against intestinal inflammation and reduced the changes in the gut microbiome (189). LGG also attenuated bone loss in ovariectomy-induced postmenopausal mice models. Inhibition of bone loss was due to increased anti-osteoclastogenic CD4^+^Foxp3^+^Tregs and CD8^+^Foxp3^+^Tregs and reduced bone marrow, peyer's patch, spleen, and lymph nodes levels (190).

### Liver Disease

Alcoholic liver disease is one of the major liver diseases and has increased morbidity and mortality rates. Continued alcohol consumption might cause intestinal microbiota homeostasis, intestinal tight junction barrier dysfunction, endotoxemia, and ultimately steatohepatitis. LGG and bacteria-free LGG culture supernatant have shown promising results in terms of liver protection, such as promoting intestinal epithelial integrity and protecting the intestinal barrier in both animal and human subjects. In this aspect, Zhao et al. demonstrated that supplementation with LGG (10^9^ CFU/day/mouse) decreased ethanol-elevated miR122a expression levels and attenuated ethanol-induced liver injury in mice (191).

Cholestatic liver disease is characterized by gut dysbiosis and increased toxic hepatic bile acids.

The mechanism of action by LGG on hepatic bile acids, liver injury, fibrosis, were evaluated in bile duct ligation (BDL) and multidrug resistance protein two knockout (*Mdr2*^−/−^) mice. LGG-treated mice had reduced hepatic concentration of taurine-β-muricholic acid and normalized levels of chenodeoxycholic acid compared to BDL mice. Also, the LGG-group mice had increased serum and ileum fibroblast growth factor 15 expression levels and reduced hepatic cholesterol 7α-hydroxylase (192). Further details can be seen in Table 6.

### Immunomodulatory Effects

In recent years, immunotherapy has become an increasing anti-cancer treatment due to its fewer side effects compared to conventional ones. LABs in the gut microbiome of patients under immunotherapy had favorable, responsive rates compared to patients lacking them. Viable and heat-killed LGG were administered to colonic cancer mice models to assess the gut immune background changes. LGG-supplemented rats had increased colonic CD8 T-cell (cytotoxic T lymphocytes) responses and decreased tumor burden in the murine gut cancer models by a CD8 Tcell–dependent manner (193).

Mast cells are multifunctional regulator cells responsible for defense against pathogenic microbes. Thus, the effect of LGG on human mast cell gene expression using microarray analysis was investigated. LGG suppressed genes encoded allergy-related high-affinity IgE receptor subunits α and γ (FCER1A and FCER1G) and histamine H4 receptor. Also, LGG up-regulated the expression levels of genes involved in mast cell immune system regulation and genes that encode proteins with a pro-inflammatory impact (IL-8) and with anti-inflammatory functions (IL-10) (194).

Giardiasis causes intestinal malabsorption and diarrhea in malnourished and immunodeficient individuals, but frequently in children <3 years old. Thus, the immunomodulatory effect of orally administered LGG in Giardia-infected mice was investigated. Pre-and post-probiotic oral administration for 25 days modulated the mucosal immune system response, as the levels of IgA antibody, IgA+ cells, and CD4+ T lymphocytes increased, as well as increased levels of anti-inflammatory cytokines (i.e., IL-6 and Il-10) and decreased levels of pro-inflammatory cytokine INF-γ (195).

Using several BP fermentation variants, Knazovická et al. noticed a decrease in *Enterobacteriaceae*, making the fermented products appropriate for consumption by people with lower immunity. Apart from the types of fermentation involved in the process, natural products with antibiotic attributes developed by the existing microbiota in stored BP, interactions among living microorganisms can control and enhance the microbiota (73). Furthermore, the resulting BB is more suitable for the organism due to its increased digestibility (196).

### Allergic Asthma

Asthma is an airway inflammatory disorder, and its prevalence is increasing by each year. Recent epidemiological studies demonstrated the relationship between asthma, allergic disorder development, and altered intestinal microbiota in both animal models (197–200) and human clinical trials (201–203). Intestinal colonization with freeze-dried LGG in pregnant and during lactation of BALB/c female mice for 48 days resulted in reduced allergic airway, peribronchial inflammation, goblet cell hyperplasia, and increased TNF-α levels (198). Also, pre-and post-treatment with LGG suppressed the airway hyper-responsiveness to methacholine and metalloproteinase nine expressions in lung tissue. Also, it significantly decreased the number of infiltrating inflammatory cells and Th2 cytokines in bronchoalveolar lavage fluid and serum compared with the OVA-sensitized mice (204). Recently, it was demonstrated that pre-and post-supplementation with LGG suppressed Th2 cytokine (T helper type II cells), TNF-α, IL-17, and HMGB (high mobility group box 1) in the BALF (Bronchoalveolar Lavage Fluid) levels and increased the levels of proteins involved in immune system responses, namely T-bet and Foxp3 in ARN (205).

The efficacy of LGG administration was studied on at-risk infants, as children of allergic mothers have an increased risk of developing allergies. Thus, LGG administration for 6 months diminished by almost 50% the incidence of allergy to expecting mothers and their infant's (197). No significant differences in asthma and allergic rhinitis were shown between early probiotic and placebo supplementation in infants (206).

### Atopic Dermatitis

Atopic dermatitis (AD) is an increasing chronic skin disease in children with a prevalence reaching 10% in many industrialized countries (207). The rising incidence of atopic dermatitis might be associated with shifts in gastrointestinal microbiota, namely from a rich flora in non-pathogenic bacteria (i.e., *Lactobacilli* or *Bifidobacterium*) to one with increased pathogenic bacteria (i.e., *Clostridium*) (208). The preventive effect of Lactobacillus on the incidence of atopic dermatitis was demonstrated in pre-and postnatal infants who were less likely to develop this disease compared to placebo group infants. In a randomized, double-blind trial, pregnant women with atopic eczema, allergic rhinitis, or asthma received LGG (1 × 10^10^ CFU/day) between 2 and 4 weeks before the estimated delivery date. After delivery, infants received LGG for 6 months. In the 4^th^ year, at the end of the trial, 46% of the children in the placebo group developed AD, compared to 26% in the probiotic group (201, 209). In a subsequent randomized, placebo-controlled trial, the children completing the previous studies were invited to complete a 7-year treatment. The LGG group had a lower risk of developing eczema (42%) compared to the placebo group (66%) (210). Rautava, Kalliomäki, and Isolauri in 2002 found that the levels of transforming growth factor (TGF)-β was significantly higher in the breast milk of LGG-administered mothers (2 × 10^10^ CFU/day) before labor and 3 months after birth (211). Furthermore, AD development was significantly lower in infants receiving LGG in the first 2 years compared to the placebo group. In multiple studies, no significant differences were observed between the probiotic and placebo-treated patients (212–214) or in IL-4, IL-5, and interferon γ (IFN γ) (215). Also, no significant differences between the probiotic and placebo groups regarding the eczema frequencies and atopic eczema were noticed (206, 216). Recently, Filipovic et al., in 2020, demonstrated that LGG formulation with Zn and vitamin D3 supplementation in infancy and early childhood proves to be an effective treatment against AD (161).

### Lowering Cholesterol Levels

Excess cholesterol is associated with coronary artery disease, the most common cardiovascular disease with increased worldwide mortality by each year. Thus, there is a need for natural therapeutic products that lower cholesterol levels besides the existing treatment schemes. In this aspect, Kim et al. orally administered LGG to high-fat diet (HFD) mice for 13 weeks. A significant reduction of weight and adipose tissues were noticed in LGG-treated HFD-fed mice compared to control. The triglyceride serum levels and cholesterol were also significantly reduced (217).

In 2008, Vamanu et al. performed a study analyzing the evolution of *L. acidophilus* 1a, and *L. plantarum* 2s strains on a media with BP and honey. The study aimed to demonstrate the outcome of fermented products on cholesterol levels in Wistar rats (*n* = 40) and possible bio-productive effects. An increase in sugar consumption after 72 h of fermentation was noticed in the milled pollen grains groups and an aroma specific to BP and lactic acid. The results showed that increased weight was seen in all treated groups with the symbiotic product compared to control. Other monitored parameters were taste and aroma, highlighting that once lactic acid is produced, the sweet flavor converts to a mix between sweet and sour (218).

### Safety and Digestibility

Understanding intestinal health and disease is strongly linked to identifying the functional elements behind the gut epithelia's effective growth and homeostasis. Recently, it was demonstrated that the intake of LGG elevated the leptin levels in the gut epithelia important in normal metabolism function and intestinal development and triggered cell proliferation (219).

According to the results presented in previous studies, in 2011, Fuenmayor et al. assessed the conditions required for a better BP fermentation from Columbia. Their results showed the possibility to use *L. acidophilus* inoculum in a matrix of BP to induce lactic fermentation and generate a probiotic product with additional functional characteristics, which might be used as a protein food supplement suitable for human consumption or as an ingredient in the development of other functional foods (98).

The massive role of *A. kunkeei* in preserving BB has been demonstrated in previous studies that have recognized BB's stability to the influences caused by nectar, honey, bee secretions, and BP natural properties (7). The fermentation protocol developed during this study emulates the natural BB fermentation process, generating a stable and safe fermented product with increased digestibility and bioavailability of nutrients due to the use of specific strains of lactobacilli as *A. kunkeei* (45).

Confectionary foods contain large numbers of titanium dioxide nanoparticles (TiO_2_ NPs), proving to be at a high risk of developing diet-induced metabolism syndrome (MetS). In this aspect, LGG-oral supplementation to MetS mice ameliorates TiO_2_ NPs-induced inflammation and metabolic abnormality caused by fructose. In LGG-supplemented mice, the gut dysbiosis was improved, and the inflammation-related bacteria (*Clostridia, Desulfovibrionaceae*, and *Proteobacteria*) were decreased; thus LGG acted as a defensive system against TiO_2_ NPs-induced severe inflammation damage (220).

Urinary symptoms are common in people suffering from neurogenic lower urinary tract dysfunction (NLUTD). As there are no non-prescription treatment methods (221), assessed the safety and tolerability of LGG in adults and children with NLUTD due to spinal cord injury or disease. At the end of the 18-month study, two doses of self-installed intravesical LGG were safe and well-tolerated among the tested individuals. This aspect was further confirmed by LGG installation in asymptomatic patients with neuropathic bladder. No adverse effects were reported after installation (222).

## Impact Assessment of Current BB Production

Because collecting BB from the hive is time-consuming and harmful, it's marketing for human use is hampered. On the other hand, pollen removal through traps is handy for beekeepers and harmless to the hive (17). Harvesting BB can be a profitable option, but it is rarely applied in apiaries due to high costs and time-consuming. However, it is an auspicious opportunity, thanks to the rising consumer awareness of functional foods (i.e., food with positive and pro-health effects on human individuals, primarily due to the naturally present bioactive substances) (223).

The process of acquiring the final product can technically be entirely automated. However, in conventional apiaries, which are the most common in EU nations and have an average of 21 hives per beekeeper, it is not economically justified (224). A 3-year study began in the spring of 2015 to establish the volume of BB production in honeybee colonies and analyze the economic effects of such output. Each year, 28 honeybee colonies participated in the experiment, separated into four groups. Each group tried alternative brood nest configurations or frame positioning against the hive entrance for collected BB. All costs, including labor, were associated with the BB production process. Depending on the group, it gathered between 0.51 and 1.23 kg of BB from a single colony. The average yield was 0.7 kg, while the apiary as a whole produced 20 kg of BB each year.

Moreover, the annual expenditures associated with BB manufacturing were 679.5 EUR, whereas the projected sales gain was 1110 EUR. As a result, the income was 430.5 EUR or 21.5 EUR per kilogram of collected BB. The most significant expenses were associated with labor, which might be a factor restricting the growth of BB manufacturing in apiaries (49).

## Challenges and Opportunities for Developing Improved Fermented Functional Foods

Fermented foods and beverages are global edible products of significant scientific, social, and economic importance. A fermented nutritive matrix represents one-third of all foods produced for human consumption (225). Furthermore, the attention of prestigious international institutions, such as the World Health Organization (WHO) and the Food and Agriculture Organization (FAO) of the United Nations (UN), to the importance of microbiological risks assessment and of traditional fermented food and beverages contribute to testify to the relevance of the discussed topic (226). The intake of fermented foods and drinks is typically connected with beneficial properties (227). This increased nutritional and functional quality would serve as an additional reason to establish a risk management plan to minimize contamination (225). The establishment of a multi-strain starter culture based on genotypic and technical characterization of microbial diversity connected with natural food fermentation to increase the “unique characteristics” defined as fermented attributes (228). It is critical to differentiate between the risk associated with microbial genera/species that are not generally present in fermented matrices and the risks associated with spontaneous fermentation. Pathogens are classified as the first class, whereas mycotoxins, ethyl carbamate, and biogenic amines are second. Because the same species includes both technologically beneficial and harmful strains, the second category is more subtle (229). Controlling the microbiology of fermented foods may be an essential step toward harnessing the biotechnological potential of strains developed via spontaneous fermentation (230). Recent economic, productive, and social trends have rediscovered the possibility of spontaneous fermentation in increasing the uniqueness of fermented goods.

## Future Perspectives and Conclusions

Over the last decade, aspects of artificial food additives and consumer demands for balanced and healthy diets may have accelerated research on natural products, such as BB. As a result, researchers are working to uncover its nutritional importance and health-promoting effects. A comparison of expenses and earnings generated by the production of BB, obtained in nature in the hive, showed that the financial impact could sometimes be less satisfactory. Labor costs can be a problem that restricts the development of this beekeeping product, so a biotechnological approach may be a better way to improve the market's presence. In the case of fermented BP consumed by humans, the chosen starters improved the bioavailability and digestibility of nutrients and bioactive compounds naturally found in BP.

Moreover, from an economic standpoint, the biotechnological development of BB may be an alternative and efficient way to diversify the beekeeping activity, which is gradually susceptible to frequent problems and increasing beekeepers' incomes. Further research into the classification of aromatic compounds, the flavor impact of organic acid-aroma interactions, chosen LAB starters and fermentation protocols, as well as consumer demands, will lead to noteworthy advancements in the flavor of fermented bee products for a promising market. Additionally, the need for value-added products with numerous health properties, macro-, and micronutrients, important bio-elements, probiotics, and vitamins is gaining popularity.

## Author Contributions

DGB and MC-C: conceptualization, methodology, and writing—original draft preparation. RM: validation, supervision, and writing—review & editing. DCV: funding acquisition, supervision, and writing—review and editing. All authors read and approved the manuscript.

## Funding

This work was supported by the National Research, Development, and Innovations Programme for 2015-2020-PNII, developed with the support of UEFISCDI PN-III-P1-1.1-TE-2019-1748 (TE 184/2020), and the publication was supported by funds from the National Research Development Projects to finance excellence (PFE)-14/2022-2024 granted by the Romanian Ministry of Research and Innovation.

## Conflict of Interest

The authors declare that the research was conducted in the absence of any commercial or financial relationships that could be construed as a potential conflict of interest.

## Publisher's Note

All claims expressed in this article are solely those of the authors and do not necessarily represent those of their affiliated organizations, or those of the publisher, the editors and the reviewers. Any product that may be evaluated in this article, or claim that may be made by its manufacturer, is not guaranteed or endorsed by the publisher.

## References

[1] Mărgăoan R Stran? M Varadi A Topal E Yücel B Cornea-Cipcigan M. Bee collected pollen and bee bread: bioactive constituents and health benefits. Antioxidants. (2019) 8:568. 10.3390/antiox8120568 31756937 PMC6943659

[2] Kieliszek M Piwowarek K Kot AM Błazejak S Chlebowska-Smigiel A Wolska I. Pollen and bee bread as new health-oriented products: A review. Trends Food Sci Technol. (2018) 71:170–80. 10.1016/j.tifs.2017.10.021

[3] Khalifa SAM Elashal M Kieliszek M Ghazala NE Farag MA Saeed A. Recent insights into chemical and pharmacological studies of bee bread. Trends Food Sci Technol. (2020) 97:300–16. 10.1016/j.tifs.2019.08.021

[4] Mărgăoan R Topal E Balkanska R Yücel B Oravecz T Cornea-Cipcigan M. Monofloral honeys as a potential source of natural antioxidants, minerals and medicine. Antioxidants. (2021) 10:1023. 10.3390/antiox10071023 34202118 PMC8300703

[5] Mărgăoan R Özkök A Keskin S Mayda N Urcan AC Cornea-Cipcigan M. Bee collected pollen as a value-added product rich in bioactive compounds and unsaturated fatty acids: A comparative study from Turkey and Romania. LWT. (2021) 149:111925. 10.1016/j.lwt.2021.111925

[6] Mărgăoan R Zăhan M Mărghitaş LA Dezmirean DS Erler S Bobiş O. Antiproliferative activity and apoptotic effects of *Filipendula ulmaria* pollen against C26 mice colon tumour cells. J Apicult Sci. (2016) 60:135–44. 10.1515/jas-2016-0014

[7] Anderson KE Carroll MJ Sheehan T Mott BM Maes P Corby-Harris V. Hive-stored pollen of honey bees: many lines of evidence are consistent with pollen preservation, not nutrient conversion. Mol Ecol. (2014) 23:5904–17. 10.1111/mec.12966 25319366 PMC4285803

[8] Degrandi-Hoffman G Chen Y Simonds R. The effects of pesticides on queen rearing and virus titers in honey bees (*Apis mellifera* L.). Insects. (2013) 4:71–89. 10.3390/insects4010071 26466796 PMC4553430

[9] Siksna S Daberte I Barene I. Investigation of bee bread and development of its dosage forms. Medicinos teorija ir praktika. (2014) 21:16–22. 10.15591/mtp.2015.003

[10] Celina Habryka Marek Kruczek Drygaś B. Bee Products Used in Apitherapy. Darwin, Poland: Scientific Publishing House. (2016).

[11] Dranca F Ursachi F Oroian M. Bee bread: physicochemical characterization and phenolic content extraction optimization. Foods. (2020) 9:1358. 10.3390/foods9101358 32987873 PMC7599645

[12] Urcan AC Marghitas LA Dezmirean DS Bobis O Bonta V Muresan CI. Chemical composition and biological activities of beebread – review. Bull University Agri Sci Vet Med Cluj-Napoca Animal Sci Biotechnol. (2017) 74:6. 10.15835/buasvmcn-asb:12646

[13] Bakour M Al-Waili NS El Menyiy N Imtara H Figuira AC Al-Waili T. Antioxidant activity and protective effect of bee bread (honey and pollen) in aluminum-induced anemia, elevation of inflammatory makers and hepato-renal toxicity. J Food Sci Technol. (2017) 54:4205–12. 10.1007/s13197-017-2889-9 29184226 PMC5686000

[14] Bakour M Fernandes Â Barros L Sokovic M Ferreira ICFR Badiaa L. Bee bread as a functional product: Chemical composition and bioactive properties. LWT. (2019) 109:276–82. 10.1016/j.lwt.2019.02.008

[15] Medina Á González G Sáez JM Mateo R Jiménez M. Bee Pollen, a Substrate that Stimulates Ochratoxin A Production by *Aspergillus ochraceus* Wilh. Syst Appl Microbiol. (2004) 27:261–7. 10.1078/072320204322881880 15046315

[16] González G Hinojo MJ Mateo R Medina A Jiménez M. Occurrence of mycotoxin producing fungi in bee pollen. Int J Food Microbiol. (2005) 105:1–9. 10.1016/j.ijfoodmicro.2005.05.001 16009441

[17] Nardoni S D'Ascenzi C Rocchigiani G Moretti V Mancianti F. Occurrence of moulds from bee pollen in Central Italy – A preliminary study. Ann Agri Environ Med. (2015) 23:103–5. 10.5604/12321966.1196862 27010410

[18] Carlos M Zuluaga JCS Marta C. Chemical, Nutritional and Bioactive Characterization of Colombian Bee-Bread (2015).

[19] Karaçil MS Tek NA. Dünyada Üretilen Fermente Ürünler: Tarihsel Süreç ve Saglik ile Ilişkileri. (2013).

[20] Kaškoniene V Adaškevičiute V Kaškonas P Mickiene R Maruška A. Antimicrobial and antioxidant activities of natural and fermented bee pollen. Food Biosci. (2020) 34:100532. 10.1016/j.fbio.2020.100532

[21] Salazar-González C Díaz-Moreno C. The nutritional and bioactive aptitude of bee pollen for a solid-state fermentation process. J Apicult Res. (2016) 55:161–75. 10.1080/00218839.2016.1205824

[22] Aylanc V Falcão SI Ertosun S Vilas-Boas M. From the hive to the table: Nutrition value, digestibility and bioavailability of the dietary phytochemicals present in the bee pollen and bee bread. Trends Food Sci Technol. (2021) 109:464–81. 10.1016/j.tifs.2021.01.042

[23] Tomás A Falcão SI Russo-Almeida P Vilas-Boas M. Potentialities of beebread as a food supplement and source of nutraceuticals: Botanical origin, nutritional composition and antioxidant activity. J Apicultural Res. (2017) 56:219–30. 10.1080/00218839.2017.1294526

[24] Filannino P Di Cagno R Addante R Pontonio E Gobbetti M. Metabolism of fructophilic lactic acid bacteria isolated from the *Apis mellifera* L. bee gut: phenolic acids as external electron acceptors. Appl Environ Microbiol. (2016) 82:6899–911. 10.1128/AEM.02194-16 27637884 PMC5103089

[25] Bojan A Goran J Mića M Jordan M Mirjana P Nedić N. Quality of pollen and honey bee bread collected in spring. J Hyg Eng Des. (2012).

[26] Donkersley P Rhodes G Pickup RW Jones KC Wilson K. Honeybee nutrition is linked to landscape composition. Ecol Evol. (2014) 4:4195–206. 10.1002/ece3.1293 25505544 PMC4242570

[27] Andjelković MS Petrović V Stamenković Z Ristić GS Jovanović GS. Circuit-level simulation of the single event transients in an on-chip single event latchup protection switch. J Electr Test. (2015) 31:275–89. 10.1007/s10836-015-5529-1

[28] Fatrcová-Šramková K NôŽková J Kačániová M Máriássyová M Rovná K Stričík M. Antioxidant and antimicrobial properties of monofloral bee pollen. J Environ Sci Health Part B Pesticides Food Contaminants Agri Wastes. (2013) 48:133–8. 10.1080/03601234.2013.727664 23305281

[29] Degrandi-Hoffman G Eckholm BJ Huang MH. A comparison of bee bread made by Africanized and European honey bees (*Apis mellifera*) and its effects on hemolymph protein titers. Apidologie. (2013) 44:52–63. 10.1007/s13592-012-0154-9

[30] Bayram NE Gercek YC Çelik S Mayda N Kostić AŽ Dramićanin AM. Phenolic and free amino acid profiles of bee bread and bee pollen with the same botanical origin – similarities and differences. Arab J Chem. (2021) 14:103004. 10.1016/j.arabjc.2021.103004

[31] Venskutonis PR Kaškoniene V Račys J Ceksteryte V. Fatty acid composition in beebread. Biologija. (2008) 54:253–7. 10.2478/v10054-008-0052-2

[32] Mărgăoan R Chirilă F. Bee Pollen Methanolic Extracts: Total Polyphenols Content and Antibacterial Activity. (2021).

[33] Pascoal A Rodrigues S Teixeira A Feás X Estevinho LM. Biological activities of commercial bee pollens: Antimicrobial, antimutagenic, antioxidant and anti-inflammatory. Food Chem Toxicol. (2014) 63:233–9. 10.1016/j.fct.2013.11.010 24262487

[34] Ivanišová E Kačániová M Frančáková H Petrová J Hutková J Brovarskyi V. Bee bread - perspective source of bioactive compounds for future. Potravinarstvo. (2015) 9:558. 10.5219/558

[35] Sawicki T Baczek N Starowicz M. Characterisation of the total phenolic, vitamins C and E content and antioxidant properties of the beebread and honey from the same batch. Czech J Food Sci. (2020) 38:158–63. 10.17221/312/2019-CJFS

[36] Sattler JAG De-Melo AAM Nascimento KSD Melo ILPD Mancini-Filho J Sattler A. Essential minerals and inorganic contaminants (barium, cadmium, lithium, lead and vanadium) in dried bee pollen produced in Rio Grande do Sul State, Brazil. Food Sci Technol. (2016) 36:505–9. 10.1590/1678-457X.0029

[37] Nakajima Y Tsuruma K Shimazawa M Mishima S Hara H. Comparison of bee products based on assays of antioxidant capacities. BMC Complement Alternative Med. (2009) 9:4. 10.1186/1472-6882-9-4 19243635 PMC2664783

[38] Veiga RS De Mendonça S Mendes PB Paulino N Mimica MJ Lagareiro Netto AA. Artepillin C and phenolic compounds responsible for antimicrobial and antioxidant activity of green propolis andBaccharis dracunculifoliaDC. J Appl Microbiol. (2017) 122:911–20. 10.1111/jam.13400 28066967

[39] Yildirim A Duran GG Duran N Jenedi K Bolgul BS Miraloglu M. Antiviral activity of hatay propolis against replication of herpes simplex virus type 1 and type 2. Med Sci Monit. (2016) 22:422–30. 10.12659/MSM.897282 26856414 PMC4750782

[40] Rimbach G Fischer A Schloesser A Jerz G Ikuta N Ishida Y. Anti-inflammatory properties of Brazilian green propolis encapsulated in a γ-cyclodextrin complex in mice fed a western-type diet. Int J Mol Sci. (2017) 18:1141. 10.3390/ijms18061141 28587122 PMC5485965

[41] Liu C-C Hao D-J Zhang Q An J Zhao J-J Chen B. Application of bee venom and its main constituent melittin for cancer treatment. Cancer Chemother Pharmacol. (2016) 78:1113–30. 10.1007/s00280-016-3160-1 27677623

[42] Atkin SL Barrier S Cui Z Fletcher PDI Mackenzie G Panel V. UV and visible light screening by individual sporopollenin exines derived from *Lycopodium clavatum* (club moss) and *Ambrosia trifida* (giant ragweed). J Photochem Photobiol B. (2011) 102:209–17. 10.1016/j.jphotobiol.2010.12.005 21232973

[43] Gilliam M Roubik D Lorenz B. Microorganisms associated with pollen, honey, and brood provisions in the nest of a stingless bee, *Melipona fasciata*. Apidologie. (1990) 21:89–97. 10.1051/apido:19900201

[44] Gilliam M Buchmann SL Lorenz BJ Roubik DWJB. Microbiology of the larval provisions of the stingless bee, *Trigona hypogea*, an obligate necrophage. Biotropica. (1985) 17:28. 10.2307/2388374

[45] Di Cagno R Filannino P Cantatore V Gobbetti M. Novel solid-state fermentation of bee-collected pollen emulating the natural fermentation process of bee bread. Food Microbiol. (2019) 82:218–30. 10.1016/j.fm.2019.02.007 31027777

[46] Tamarit D Ellegaard KM Wikander J Olofsson T Vasquez A Andersson SGE. Functionally structured genomes in *Lactobacillus kunkeei* colonizing the honey crop and food products of honeybees and stingless bees. Genome Biol Evol. (2015) 7:1455–73. 10.1093/gbe/evv079 25953738 PMC4494060

[47] Mayda N Ozkok A Bayram NE Gercek YC Sorkun K. Bee bread and bee pollen of different plant sources: determination of phenolic content, antioxidant activity, fatty acid and element profiles. J Food Meas Charact. (2020) 14:1795–809. 10.1007/s11694-020-00427-y

[48] Souza RCdS Yuyama LKO Aguiar JPL Oliveira FPM. Valor nutricional do mel e pólen de abelhas sem ferrão da região amazônica. (2014).

[49] Semkiw P Skubida P. Bee bread production—a new source of income for beekeeping farms? Agriculture. (2021) 11:6. 10.3390/agriculture11060468

[50] Mărgăoan R Cornea-Cipcigan M Topal E Kösoglu M. Impact of fermentation processes on the bioactive profile and health-promoting properties of bee bread, mead and honey vinegar. Processes. (2020) 8:1081. 10.3390/pr8091081

[51] Khalid K. An Overview of Lactic Acid Bacteria. (2011).

[52] Waśko A Kieliszek M Targoński Z. Purification and characterization of a proteinase from the probiotic *Lactobacillus rhamnosu*s OXY. Preparative Biochem Biotechnol. (2012) 42:476–88. 10.1080/10826068.2012.656869 22897769

[53] García C Rendueles M Díaz M. Liquid-phase food fermentations with microbial consortia involving lactic acid bacteria: A review. Food Res Int. (2019) 119:207–20. 10.1016/j.foodres.2019.01.043 30884650

[54] Florou-Paneri P Christaki E Bonos E. Lactic acid bacteria as source of functional ingredients. InTech. (2013) 25:589–614. 10.5772/47766

[55] Peng K Koubaa M Bals O Vorobiev E. Recent insights in the impact of emerging technologies on lactic acid bacteria: A review. Food Res Int. (2020) 137:109544. 10.1016/j.foodres.2020.109544 33233170

[56] Mora WI Fuenmayor CA Benavides MA Algecira NA Quicazán MC. Bee pollen as a novel substrate in pilot-scale probiotic-mediated lactic fermentation processes. LWT. (2021) 2021:110868. 10.1016/j.lwt.2021.110868

[57] Koch H Schmid-Hempel P. Bacterial communities in central european bumblebees: low diversity and high specificity. Microbial Ecol. (2011) 62:121–33. 10.1007/s00248-011-9854-3 21556885

[58] Van De Guchte M Penaud S Grimaldi C Barbe V Bryson K Nicolas P. The complete genome sequence of *Lactobacillus bulgaricus* reveals extensive and ongoing reductive evolution. Proc Natl Acad Sci USA. (2006) 103:9274–9. 10.1073/pnas.0603024103 16754859 PMC1482600

[59] Endo A Maeno S Tanizawa Y Kneifel W Arita M Dicks L. Fructophilic lactic acid bacteria, a unique group of fructose-fermenting microbes. Appl Environ Microbiol. (2018) 84:19. 10.1128/AEM.01290-18 30054367 PMC6146980

[60] Endo A Tanizawa Y Tanaka N Maeno S Kumar H Shiwa Y. Comparative genomics of *Fructobacillus spp*. and Leuconostoc spp. reveals niche-specific evolution of Fructobacillus spp. BMC Genomics. (2015) 16:1117. 10.1186/s12864-015-2339-x 26715526 PMC4696137

[61] Zaunmüller T Eichert M Richter H Unden G. Variations in the energy metabolism of biotechnologically relevant heterofermentative lactic acid bacteria during growth on sugars and organic acids. Appl Microbiol Biotechnol. (2006) 72:421–9. 10.1007/s00253-006-0514-3 16826375

[62] Calasso M Gobbetti M. Lactic acid bacteria | Lactobacillus spp.: other species. Encyclopedia of Dairy Sciences. Amsterdam: Elsevier Ltd. (2011). 10.1016/B978-0-12-374407-4.00265-X

[63] Batt CA. Lactobacillus | Introduction. Encyclopedia Food Microbiol. (2014) 2014:409–11. 10.1016/B978-0-12-384730-0.00176-2

[64] Filannino P Di Cagno R Gambacorta G Tlais AZ Cantatore V Gobbetti M. Volatilome and bioaccessible phenolics profiles in lab-scale fermented bee pollen. Foods. (2021) 10:20286. 10.3390/foods10020286 33572637 PMC7911640

[65] Abushelaibi A Al-Mahadin S El-Tarabily K Shah NP Ayyash M. Characterization of potential probiotic lactic acid bacteria isolated from camel milk. LWT. (2017) 79:316–25. 10.1016/j.lwt.2017.01.04131672637

[66] Mašková Z Knazovická V Tančinová D Panáková S. Production of pollen cans by fermentation of bee pollen in model conditions with regard to filamentous micromycetes occurrence. J Microbiol Biotechnol Food Sci. (2019) 8:1223–7. 10.15414/jmbfs.2019.8.5.1223-1227

[67] Zuluaga-Dominguez CM Quicazan M. Effect of fermentation on structural characteristics and bioactive compounds of bee-pollen based food. J Apicultural Sci. (2019) 63:209–22. 10.2478/jas-2019-0016

[68] Araneda X Velásquez C Morales D Martínez I. Producción de pan de abejas (*Apis mellifera* L.) bajo condiciones de laboratorio. Idesia (Arica). (2014) 32:63–9. 10.4067/S0718-34292014000400008

[69] Risco-Ríos CAd Pérez-Piñeiro A Álvarez-Rivera VP RodríguezCastro i Virginia Leiva-Castillo Puig-Peña Y. Bacterias ácido-lácticas para ensilar polen apícola. (2012).

[70] Endo A Futagawa-Endo Y Dicks LMT. Isolation and characterization of fructophilic lactic acid bacteria from fructose-rich niches. Syst Appl Microbiol. (2009) 32:593–600. 10.1016/j.syapm.2009.08.002 19733991

[71] Yang S Wu Y Luo C Di Z Wu Y Zhang J. A *Bacillus coagulans* and its application in pine pollen fermentation. Food Sci Biotechnol. (2015) 24:2129–35. 10.1007/s10068-015-0283-9

[72] Mohr KI Tebbe CC. Diversity and phylotype consistency of bacteria in the guts of three bee species (Apoidea) at an oilseed rape field. Environ Microbiol. (2006) 8:258–72. 10.1111/j.1462-2920.2005.00893.x 16423014

[73] Knazovická V Mašková Z Vlková E Švejstil R Salmonová H Ivanišová E. Pollen can - testing of bee pollen fermentation in model conditions. J Microbiol Biotechnol Food Sci. (2018) 8:805–11. 10.15414/jmbfs.2018.8.2.805-811

[74] Vamanu A Vamanu E Popa O Câmpeanu G Albulescu R Drugulescu M. Obtaining of a symbiotic product based on lactic bacteria, pollen and honey. Pakistan J Biol Sci. (2008) 11:613–7. 10.3923/pjbs.2008.613.617 18817135

[75] Linjordet MS. A Comparative Analysis of Lactic Acid Bacteria Isolated from Honeybee Gut and Flowers, with Focus on Phylogeny and Plasmid Profile. Ås: Norwegian University of Life Sciences. (2016).

[76] Yang S Li H. Optimization of pine pollen fermentation conditions using *Lactobacillus paracasei*. Food Sci Biotechnol. (2015) 24:155–60. 10.1007/s10068-015-0021-3

[77] du Plessis HW Dicks LMT Pretorius IS Lambrechts MG du Toit M. Identification of lactic acid bacteria isolated from South African brandy base wines. Int J Food Microbiol. (2004) 91:19–29. 10.1016/S0168-1605(03)00335-0 14967557

[78] Endo A Tanaka N Oikawa Y Okada S Dicks L. Fructophilic Characteristics of *Fructobacillus spp*. may be due to the Absence of an Alcohol/Acetaldehyde Dehydrogenase Gene (adhE). Curr Microbiol. (2014) 68:531–5. 10.1007/s00284-013-0506-3 24352296

[79] Vamanu E Vamanu A Popa O Babeanu NJSPASB. The antioxidant effect of a functional product based on probiotic biomass, pollen and honey. Animal Sci Biotechnol. (2010) 43:331–6. Available online at: https://www.usab-tm.ro/fileadmin/fzb/Simp%202010/vol1/BIOTECHNOLOGIES/Vamanu1.pdf

[80] Vergalito F Testa B Cozzolino A Letizia F Succi M Lombardi SJ. Potential application of *Apilactobacillus kunkeei* for human use: evaluation of probiotic and functional properties. Foods. (2020) 9:1535. 10.3390/foods9111535 33113800 PMC7693146

[81] CadeŽ N Fülöp L Dlauchy D Péter G. *Zygosaccharomyces favi sp. nov.*, an obligate osmophilic yeast species from bee bread and honey. Antonie van Leeuwenhoek. (2015) 107:645–54. 10.1007/s10482-014-0359-1 25528339

[82] Iorizzo M Pannella G Lombardi SJ Ganassi S Testa B Succi M. Inter- and intra-species diversity of lactic acid bacteria in Apis mellifera ligustica colonies. Microorganisms. (2020) 8:1578. 10.3390/microorganisms8101578 33066358 PMC7602248

[83] Endo A Okada S. Reclassification of the genus Leuconostoc and proposals of *Fructobacillus fructosus* gen. *nov., comb. nov., Fructobacillus durionis comb. nov., Fructobacillus ficulneus comb. nov. and Fructobacillus pseudoficulneus comb. nov*. Int J Syst Evol Microbiol. (2008) 58:2195–205. 10.1099/ijs.0.65609-0 18768629

[84] Tang QH Miao CH Chen YF Dong ZX Cao Z Liao SQ. The composition of bacteria in gut and beebread of stingless bees (Apidae: *Meliponini*) from tropics Yunnan, China. Antonie Van Leeuwenhoek. (2021) 114:1293–305. 10.1007/s10482-021-01602-x 34110551

[85] Ngalimat MS Raja Abd. Rahman RNZ Yusof MT Syahir A Sabri S. Characterisation of bacteria isolated from the stingless bee, *Heterotrigona itama*, honey, bee bread and propolis. PeerJ. (2019) 7:e7478. 10.7717/peerj.7478 31497388 PMC6708576

[86] Mohammad SM Mahmud-Ab-Rashid N-K Zawawi N. Probiotic properties of bacteria isolated from bee bread of stingless bee *Heterotrigona itama*. J Apicultural Res. (2021) 60:172–87. 10.1080/00218839.2020.1801152

[87] Ramos OY Basualdo M Libonatti C Vega MF. Current status and application of lactic acid bacteria in animal production systems with a focus on bacteria from honey bee colonies. J Appl Microbiol. (2020) 128:1248–60. 10.1111/jam.14469 31566847

[88] Martinson VG Danforth BN Minckley RL Rueppell O Tingek S Moran NA. A simple and distinctive microbiota associated with honey bees and bumble bees. Mol Ecol. (2011) 20:619–28. 10.1111/j.1365-294X.2010.04959.x 21175905

[89] Shah NP. *Bifidobacterium spp*.: morphology and physiology. (2011). 10.1016/B978-0-12-374407-4.00043-1

[90] Pokusaeva K Fitzgerald GF Van Sinderen D. Carbohydrate metabolism in Bifidobacteria. Genes Nutr. (2011) 6:285–306. 10.1007/s12263-010-0206-6 21484167 PMC3145055

[91] Van Der Meulen R Adriany T Verbrugghe K De Vuyst L. Kinetic analysis of bifidobacterial metabolism reveals a minor role for succinic acid in the regeneration of NAD+ through its growth-associated production. Appl Environ Microbiol. (2006) 72:5204–10. 10.1128/AEM.00146-06 16885266 PMC1538715

[92] Emanuel Vamanu Vamanu A Popa O Băbeanu N. The Antioxidant Effect of a Functional Product Based on Probiotic Biomass, Pollen and Honey. (2010).

[93] Duan C Feng Y Zhou H Xia X Shang Y Cui Y. Optimization of fermentation condition of man-made bee-bread by response surface methodology. Adv Appl Biotechnol. (2015) 2015:353–63. 10.1007/978-3-662-46318-5_38

[94] Calasso M Gobbetti M. Lactobacillus spp.: Other Species. (2002).

[95] Zheng J Wittouck S Salvetti E Franz C Harris HMB Mattarelli P. A taxonomic note on the genus Lactobacillus: Description of 23 novel genera, emended description of the genus *Lactobacillus Beijerinck* 1901 and union of *Lactobacillaceae* and *Leuconostocaceae*. Int J Syst Evol Microbiol. (2020) 70:2782–858. 10.1099/ijsem.0.004107 32293557

[96] Yan S Li Q Xue X Wang K Zhao L Wu L. Analysis of improved nutritional composition of bee pollen (*Brassica campestris* L.) after different fermentation treatments. Int J Food Sci Technol. (2019) 54:2169–81. 10.1111/ijfs.14124

[97] Kaškoniene V Katilevičiute A Kaškonas P Maruška A. The impact of solid-state fermentation on bee pollen phenolic compounds and radical scavenging capacity. Chem Papers. (2018) 72:2115–20. 10.1007/s11696-018-0417-7

[98] Zhao Y Liu S Tang Y You T Xu H. Lactobacillus rhamnosus GG ameliorated long-term exposure to TiO(2) nanoparticles induced microbiota-mediated liver and colon inflammation and fructose-caused metabolic abnormality in metabolism syndrome mice. J Agri Food Chem. (2021) 69:9788–99. 10.1021/acs.jafc.1c03301 34382390

[99] Zhang Z Cao H Chen C Chen X Wei Q Zhao F. Effects of fermentation by *Ganoderma lucidum* and *Saccharomyces cerevisiae* on rape pollen morphology and its wall. J Food Sci Technol. (2017) 54:4026–34. 10.1007/s13197-017-2868-1 29085145 PMC5643820

[100] Vina I Semjonovs P Linde R Denina I. Current evidence on physiological activity and expected health effects of kombucha fermented beverage. J Med Food. (2013) 17:179–88. 10.1089/jmf.2013.0031 24192111

[101] Jayabalan R Malbaša RV Lončar ES Vitas JS Sathishkumar M. A review on kombucha tea—microbiology, composition, fermentation, beneficial effects, toxicity, and tea fungus. Comprehen Rev Food Sci Food Safety. (2014) 13:538–50. 10.1111/1541-4337.12073 33412713

[102] Özkaya H Özkaya B Duman B Turksoy S. Effect of dephytinization by fermentation and hydrothermal autoclaving treatments on the antioxidant activity, dietary fiber, and phenolic content of oat bran. J Agri Food Chem. (2017) 65:5713–9. 10.1021/acs.jafc.7b01698 28651042

[103] Zhou J Qi Y Ritho J Zhang Y Zheng X Wu L. Flavonoid glycosides as floral origin markers to discriminate of unifloral bee pollen by LC–MS/MS. Food Control. (2015) 57:54–61. 10.1016/j.foodcont.2015.03.035

[104] De-Melo AAM Estevinho MLMF Almeida-Muradian LB. A diagnosis of the microbiological quality of dehydrated bee-pollen produced in Brazil. Lett Appl Microbiol. (2015) 61:477–83. 10.1111/lam.12480 26280091

[105] Ekinci R. The effect of fermentation and drying on the water-soluble vitamin content of tarhana, a traditional Turkish cereal food. Food Chem. (2005) 90:127–32. 10.1016/j.foodchem.2004.03.036

[106] Chitte RR Dey S. Production of a fibrinolytic enzyme by thermophilic *Streptomyces* species. World J Microbiol Biotechnol. (2002) 18:289. 10.1023/A:1015252607118

[107] Peng Y Huang Q Zhang R-H Zhang Y-Z. Purification and characterization of a fibrinolytic enzyme produced by *Bacillus amyloliquefaciens* DC-4 screened from douchi, a traditional Chinese soybean food. Comparat Biochem Physiol Part B: Biochem Mol Biol. (2003) 134:45–52. 10.1016/S1096-4959(02)00183-5 12524032

[108] Sumi H Hamada H Tsushima H Mihara H Muraki H. A novel fibrinolytic enzyme (nattokinase) in the vegetable cheese Natto; a typical and popular soybean food in the Japanese diet. Experientia. (1987) 43:1110–1. 10.1007/BF01956052 3478223

[109] Amores-Arrocha A Roldán A Jiménez-Cantizano A Caro I Palacios V. Effect on white grape must of multiflora bee pollen addition during the alcoholic fermentation process. Molecules. (2018) 23:1321. 10.3390/molecules23061321 29857507 PMC6100549

[110] Kostić AŽ Petrović TS Krnjaja VS Nedić NM Tešić ŽL Milojković-Opsenica DM. Mold/aflatoxin contamination of honey bee collected pollen from different Serbian regions. J Apicultural Res. (2016) 56:13–20. 10.1080/00218839.2016.1259897

[111] Kostić AŽ Milinčić DD Barać MB Ali Shariati M Tešić ŽL Pešić MB. The application of pollen as a functional food and feed ingredient—the present and perspectives. Biomolecules. (2020) 10:84. 10.3390/biom10010084 31948037 PMC7023195

[112] Asama T Arima TH Gomi T Keishi T Tani H Kimura Y. *Lactobacillus kunkeei*YB38 from honeybee products enhances IgA production in healthy adults. J Appl Microbiol. (2015) 119:818–26. 10.1111/jam.12889 26121394

[113] Isayenko OY Knysh OV Babych YM Ryzhkova TN Dyukareva GI. Effect of disintegrates and metabolites of *Lactobacillus rhamnosus* and *Saccharomyces boulardii* on biofilms of antibiotic resistant conditionally pathogenic and pathogenic bacteria. Regul Mechan Biosyst. (2019) 10:3–8. 10.15421/021901

[114] Detry R Simon-Delso N Bruneau E Daniel H-M. Specialisation of yeast genera in different phases of bee bread maturation. Microorganisms. (2020) 8:1789. 10.3390/microorganisms8111789 33202620 PMC7696220

[115] Khailova L Dvorak K Arganbright KM Halpern MD Kinouchi T Yajima M. *Bifidobacterium bifidum* improves intestinal integrity in a rat model of necrotizing enterocolitis. Am J Physiol Gastrointest Liver Physiol. (2009) 297:G940–9. 10.1152/ajpgi.00141.2009 20501441 PMC2777452

[116] Türkel S Ener B. Isolation and Characterization of New *Metschnikowia pulcherrima* Strains as Producers of the Antimicrobial Pigment Pulcherrimin. Zeitschrift für Naturforschung C. (2009) 64:405–10. 10.1515/znc-2009-5-618 19678547

[117] Allonsius CN van den Broek MFL De Boeck I Kiekens S Oerlemans EFM Kiekens F. Interplay between *Lactobacillus rhamnosus* GG and Candida and the involvement of exopolysaccharides. Microbial Biotechnol. (2017) 10:1753–63. 10.1111/1751-7915.12799 28772020 PMC5658588

[118] Songkun S Shenglu C Xuping Y Xuezhen L Fuliang H. The influence of bacteria isolated from bee bread on pH. Apiculture of China. (2002) 53: 4–5.

[119] Cozzolino A Vergalito F Tremonte P Iorizzo M Lombardi SJ Sorrentino E. Preliminary evaluation of the safety and probiotic potential of *Akkermansia muciniphila* DSM 22959 in comparison with *Lactobacillus rhamnosus* GG. Microorganisms. (2020) 8:189. 10.3390/microorganisms8020189 32019075 PMC7074805

[120] Jiang Q Kainulainen V Stamatova I Korpela R Meurman JH. *Lactobacillus rhamnosus* GG in experimental oral biofilms exposed to different carbohydrate sources. Caries Res. (2018) 52:220–9. 10.1159/000479380 29353279

[121] Seow SW Cai S Rahmat JN Bay BH Lee YK Chan YH. *Lactobacillus rhamnosus* GG induces tumor regression in mice bearing orthotopic bladder tumors. Cancer Sci. (2010) 101:751–8. 10.1111/j.1349-7006.2009.01426.x 20015287 PMC11159805

[122] Linsalata M Cavallini A Messa C Orlando A Refolo MG Russo F. *Lactobacillus rhamnosus* GG influences polyamine metabolism in HGC-27 gastric cancer cell line: a strategy toward nutritional approach to chemoprevention of gastric cance. Curr Pharmaceut Design. (2010) 16:847–53. 10.2174/138161210790883598 20388096

[123] Rokka S Myllykangas S Joutsjoki V. Effect of specific colostral antibodies and selected lactobacilli on the adhesion of *Helicobacter pylori* on AGS cells and the *Helicobacter*-induced IL-8 production. Scand J Immunol. (2008) 68:280–6. 10.1111/j.1365-3083.2008.02138.x 18627549

[124] Orlando A Refolo MG Messa C Amati L Lavermicocca P Guerra V. Antiproliferative and proapoptotic effects of viable or heat-killed *Lactobacillus paracasei* IMPC2.1 and *Lactobacillus rhamnosus* GG in HGC-27 gastric and DLD-1 colon cell lines. Nutr Cancer. (2012) 64:1103–11. 10.1080/01635581.2012.717676 23061912

[125] Russo F Orlando A Linsalata M Cavallini A Messa C. Effects of *Lactobacillus rhamnosus* GG on the cell growth and polyamine metabolism in HGC-27 human gastric cancer cells. Nutr Cancer. (2007) 59:106–14. 10.1080/01635580701365084 17927509

[126] Lopez M Li N Kataria J Russell M Neu J. Live and ultraviolet-inactivated *Lactobacillus rhamnosus* GG decrease flagellin-induced interleukin-8 production in Caco-2 cells. J Nutr. (2008) 138:2264–8. 10.3945/jn.108.093658 18936229

[127] Escamilla J Lane MA Maitin V. Cell-free supernatants from probiotic *Lactobacillus casei* and *Lactobacillus rhamnosus* GG decrease colon cancer cell invasion *in vitro*. Nutr Cancer. (2012) 64:871–8. 10.1080/01635581.2012.700758 22830611

[128] Prete R Garcia-Gonzalez N Di Mattia CD Corsetti A Battista N. Food-borne *Lactiplantibacillus plantarum* protect normal intestinal cells against inflammation by modulating reactive oxygen species and IL-23/IL-17 axis. Sci Rep. (2020) 10:16340. 10.1038/s41598-020-73201-1 33004903 PMC7529774

[129] Toki S Kagaya S Shinohara M Wakiguchi H Matsumoto T Takahata Y. *Lactobacillus rhamnosus* GG and *Lactobacillus casei* suppress *Escherichia coli*-induced chemokine expression in intestinal epithelial cells. Int Arch Allergy Immunol. (2009) 148:45–58. 10.1159/000151505 18716403

[130] Uribe G Villéger R Bressollier P Dillard RN Worthley DL Wang TC. *Lactobacillus rhamnosus* GG increases cyclooxygenase-2 expression and prostaglandin E2 secretion in colonic myofibroblasts via a MyD88-dependent mechanism during homeostasis. Cell Microbiol. (2018) 20:e12871. 10.1111/cmi.12871 29920917 PMC6202218

[131] Donato KA Gareau MG Wang YJJ Sherman PM. *Lactobacillus rhamnosus* GG attenuates interferon-γ and tumour necrosis factor-α-induced barrier dysfunction and pro-inflammatory signalling. Microbiology. (2010) 156:3288–97. 10.1099/mic.0.040139-0 20656777

[132] Peña JA Versalovic J. *Lactobacillus rhamnosus* GG decreases TNF-alpha production in lipopolysaccharide-activated murine macrophages by a contact-independent mechanism. Cell Microbiol. (2003) 5:277–85. 10.1046/j.1462-5822.2003.t01-1-00275.x 12675685

[133] Wang YM Ge XZ Wang WQ Wang T Cao HL Wang BL. *Lactobacillus rhamnosus* GG supernatant upregulates serotonin transporter expression in intestinal epithelial cells and mice intestinal tissues. Neurogastroenterol Motility. (2015) 27:1239–48. 10.1111/nmo.12615 26088715

[134] Wang B Hylwka T Smieja M Surrette M Bowdish DME Loeb M. Probiotics to prevent respiratory infections in nursing homes: a pilot randomized controlled trial. J Am Geriatrics Soc. (2018) 66:1346–52. 10.1111/jgs.15396 29741754

[135] Asama T Kimura Y Kono T Tatefuji T Hashimoto K Benno Y. Effects of heat-killed *Lactobacillus kunkeei* YB38 on human intestinal environment and bowel movement: a pilot study. Benef Microbes. (2016) 7:337–44. 10.3920/BM2015.0132 26839076

[136] Asama T Uematsu T Kobayashi N Tatefuji T Hashimoto K. Oral administration of heat-killed *Lactobacillus kunkeei* YB38 improves murine influenza pneumonia by enhancing IgA production. Biosci Microbiota Food Health. (2017) 36:1–9. 10.12938/bmfh.16-010 28243545 PMC5301051

[137] Li Y-T Xu H Ye J-Z Wu W-R Shi D Fang D-Q. Efficacy of *Lactobacillus rhamnosus* GG in treatment of acute pediatric diarrhea: A systematic review with meta-analysis. World J Gastroenterol. (2019) 25:4999–5016. 10.3748/wjg.v25.i33.4999 31543689 PMC6737314

[138] Jiang Y Ye L Cui Y Yang G Yang W Wang J. Effects of *Lactobacillus rhamnosus* GG on the maturation and differentiation of dendritic cells in rotavirus-infected mice. Benef Microbes. (2017) 8:645–56. 10.3920/BM2016.0157 28670908

[139] Savino F Montanari P Galliano I Daprà V Bergallo M. *Lactobacillus rhamnosus* GG (ATCC 53103) for the management of infantile colic: a randomized controlled trial. Nutrients. (2020) 12:1693. 10.3390/nu12061693 32517123 PMC7352391

[140] Kane AF Bhatia AD Denning PW Shane AL Patel RM. Routine supplementation of *Lactobacillus rhamnosus* GG and risk of necrotizing enterocolitis in very low birth weight infants. J Pediatrics. (2018) 195:73–9.e2. 10.1016/j.jpeds.2017.11.055 29402455 PMC5869135

[141] Groele L Szajewska H Szypowska A. Effects of *Lactobacillus rhamnosus* GG and *Bifidobacterium lactis* Bb12 on beta-cell function in children with newly diagnosed type 1 diabetes: protocol of a randomised controlled trial. BMJ Open. (2017) 7:e017178. 10.1136/bmjopen-2017-017178 29025837 PMC5652563

[142] Spacova I Petrova MI Fremau A Pollaris L Vanoirbeek J Ceuppens JL. Intranasal administration of probiotic *Lactobacillus rhamnosus* GG prevents birch pollen-induced allergic asthma in a murine model. Allergy. (2019) 74:100–10. 10.1111/all.13502 29888398

[143] Han X Lee A Huang S Gao J Spence JR Owyang C. *Lactobacillus rhamnosus* GG prevents epithelial barrier dysfunction induced by interferon-gamma and fecal supernatants from irritable bowel syndrome patients in human intestinal enteroids and colonoids. Gut Microbes. (2019) 10:59–76. 10.1080/19490976.2018.1479625 30040527 PMC6363076

[144] Gatej SM Marino V Bright R Fitzsimmons TR Gully N Zilm P. Probiotic *Lactobacillus rhamnosus* GG prevents alveolar bone loss in a mouse model of experimental periodontitis. J Clin Periodontol. (2018) 45:204–12. 10.1111/jcpe.12838 29121411

[145] Sabbatini S Monari C Ballet N Decherf AC Bozza S Camilloni B. Anti-biofilm properties of *Saccharomyces cerevisiae* CNCM I-3856 and *Lacticaseibacillus rhamnosus* ATCC 53103 probiotics against G. vaginalis. Microorganisms. (2020) 8:91294. 10.3390/microorganisms8091294 32847138 PMC7564297

[146] Kumova OK Fike AJ Thayer JL Nguyen LT Mell JC Pascasio J. Lung transcriptional unresponsiveness and loss of early influenza virus control in infected neonates is prevented by intranasal *Lactobacillus rhamnosus* GG. PLoS Pathog. (2019) 15:e1008072. 10.1371/journal.ppat.1008072 31603951 PMC6808501

[147] Strauss M Mičetić-Turk D Pogačar MŠ Fijan S. Probiotics for the prevention of acute respiratory-tract infections in older people: systematic review. Healthcare. (2021) 9:690. 10.3390/healthcare9060690 34200435 PMC8228160

[148] Harata G Yoda K Wang R Miyazawa K Sato M He F. Species- and age/generation-dependent adherence of bifidobacterium bifidum to human intestinal mucus *in vitro*. Microorganisms. (2021) 9:542. 10.3390/microorganisms9030542 33808003 PMC7998455

[149] Pagnini C Corleto VD Martorelli M Lanini C D'Ambra G Di Giulio E. Mucosal adhesion and anti-inflammatory effects of *Lactobacillus rhamnosus* GG in the human colonic mucosa: A proof-of-concept study. World J Gastroenterol. (2018) 24:4652–62. 10.3748/wjg.v24.i41.4652 30416313 PMC6224475

[150] Hibberd PL Kleimola L Fiorino AM Botelho C Haverkamp M Andreyeva I. No evidence of harms of probiotic *Lactobacillus rhamnosus* GG ATCC 53103 in healthy elderly-a phase I open label study to assess safety, tolerability and cytokine responses. PLoS ONE. (2014) 9:e113456. 10.1371/journal.pone.0113456 25438151 PMC4249962

[151] Vilander AC Dean GA. Adjuvant strategies for lactic acid bacterial mucosal vaccines. Vaccines. (2019) 7:150. 10.3390/vaccines7040150 31623188 PMC6963626

[152] Ciorba MA Riehl TE Rao MS Moon C Ee X Nava GM. *Lactobacillus* probiotic protects intestinal epithelium from radiation injury in a TLR-2/cyclo-oxygenase-2-dependent manner. Gut. (2012) 61:829–38. 10.1136/gutjnl-2011-300367 22027478 PMC3345937

[153] Shi C-w Cheng M-y Yang X Lu Y-y Yin H-d Zeng Y. Probiotic *Lactobacillus rhamnosus* GG promotes mouse gut microbiota diversity and T cell differentiation. Front Microbiol. (2020) 11:e607735. 10.3389/fmicb.2020.607735 33391230 PMC7773731

[154] Zhang LY Liu S Zhao XJ Wang N Jiang X Xin HS. *Lactobacillus rhamnosus* GG modulates gastrointestinal absorption, excretion patterns, and toxicity in Holstein calves fed a single dose of aflatoxin B1. J Dairy Sci. (2019) 102:1330–40. 10.3168/jds.2018-15444 30594375

[155] Gamallat Y Meyiah A Kuugbee ED Hago AM Chiwala G Awadasseid A. *Lactobacillus rhamnosus* induced epithelial cell apoptosis, ameliorates inflammation and prevents colon cancer development in an animal model. Biomed Pharmacother. (2016) 83:536–41. 10.1016/j.biopha.2016.07.001 27447122

[156] Nermes M Kantele JM Atosuo TJ Salminen S Isolauri E. Interaction of orally administered *Lactobacillus rhamnosus* GG with skin and gut microbiota and humoral immunity in infants with atopic dermatitis. Clin Experi Allergy. (2011) 41:370–7. 10.1111/j.1365-2222.2010.03657.x 21121981

[157] Kopp MV Hennemuth I Heinzmann A Urbanek R. Randomized, double-blind, placebo-controlled trial of probiotics for primary prevention: no clinical effects of *Lactobacillus* GG supplementation. Pediatrics. (2008) 121:e850–6. 10.1542/peds.2007-1492 18332075

[158] Sawada J Morita H Tanaka A Salminen S He F Matsuda H. Ingestion of heat-treated *Lactobacillus rhamnosus* GG prevents development of atopic dermatitis in NC/Nga mice. Clin Experi Allergy. (2007) 37:296–303. 10.1111/j.1365-2222.2006.02645.x 17250703

[159] Majamaa H Isolauri E. Probiotics: a novel approach in the management of food allergy. J Allergy Clin Immunol. (1997) 99:179–85. 10.1016/S0091-6749(97)70093-9 9042042

[160] Chen RC Xu LM Du SJ Huang SS Wu H Dong JJ. *Lactobacillus rhamnosus* GG supernatant promotes intestinal barrier function, balances Treg and TH17 cells and ameliorates hepatic injury in a mouse model of chronic-binge alcohol feeding. Toxicol Lett. (2016) 241:103–10. 10.1016/j.toxlet.2015.11.019 26617183

[161] Kim B Park KY Ji Y Park S Holzapfel W Hyun CK. Protective effects of *Lactobacillus rhamnosus* GG against dyslipidemia in high-fat diet-induced obese mice. Biochem Biophys Res Commun. (2016) 473:530–6. 10.1016/j.bbrc.2016.03.107 27018382

[162] Wang Y Liu Y Sidhu A Ma Z McClain C Feng W. *Lactobacillus rhamnosus* GG culture supernatant ameliorates acute alcohol-induced intestinal permeability and liver injury. Am J Physiol Gastrointest Liver Physiol. (2012) 303:G32–41. 10.1152/ajpgi.00024.2012 22538402 PMC3404581

[163] Wang Y Liu Y Kirpich I Ma Z Wang C Zhang M. *Lactobacillus rhamnosus* GG reduces hepatic TNFα production and inflammation in chronic alcohol-induced liver injury. J Nutr Biochem. (2013) 24:1609–15. 10.1016/j.jnutbio.2013.02.001 23618528 PMC3804118

[164] Wang Y Kirpich I Liu Y Ma Z Barve S McClain CJ. *Lactobacillus rhamnosus* GG treatment potentiates intestinal hypoxia-inducible factor, promotes intestinal integrity and ameliorates alcohol-induced liver injury. Am J Pathol. (2011) 179:2866–75. 10.1016/j.ajpath.2011.08.039 22093263 PMC3260853

[165] Ritze Y Bárdos G Claus A Ehrmann V Bergheim I Schwiertz A. *Lactobacillus rhamnosus* GG protects against non-alcoholic fatty liver disease in mice. PLoS ONE. (2014) 9:e80169. 10.1371/journal.pone.0080169 24475018 PMC3903470

[166] Forsyth CB Farhadi A Jakate SM Tang Y Shaikh M Keshavarzian A. *Lactobacillus* GG treatment ameliorates alcohol-induced intestinal oxidative stress, gut leakiness, and liver injury in a rat model of alcoholic steatohepatitis. Alcohol (Fayetteville, NY). (2009) 43:163–72. 10.1016/j.alcohol.2008.12.009 19251117 PMC2675276

[167] Sindhu KN Sowmyanarayanan TV Paul A Babji S Ajjampur SS Priyadarshini S. Immune response and intestinal permeability in children with acute gastroenteritis treated with *Lactobacillus rhamnosus* GG: a randomized, double-blind, placebo-controlled trial. Clin Infect Dis. (2014) 58:1107–15. 10.1093/cid/ciu065 24501384 PMC3967829

[168] Alanzi A Honkala S Honkala E Varghese A Tolvanen M Söderling E. Effect of *Lactobacillus rhamnosus* and *Bifidobacterium lactis* on gingival health, dental plaque, and periodontopathogens in adolescents: a randomised placebo-controlled clinical trial. Benef Microbes. (2018) 9:593–602. 10.3920/BM2017.0139 29633646

[169] Polak D Wilensky A Shapira L Halabi A Goldstein D Weiss EI. Mouse model of experimental periodontitis induced by *Porphyromonas gingivalis*/*Fusobacterium nucleatum* infection: bone loss and host response. J Clin Periodontol. (2009) 36:406–10. 10.1111/j.1600-051X.2009.01393.x 19419440

[170] Wen K Tin C Wang H Yang X Li G Giri-Rachman E. Probiotic *Lactobacillus rhamnosus* GG enhanced Th1 cellular immunity but did not affect antibody responses in a human gut microbiota transplanted neonatal gnotobiotic pig model. PLoS ONE. (2014) 9:e94504. 10.1371/journal.pone.0094504 24722168 PMC3983166

[171] Capurso L. Thirty years of *Lactobacillus rhamnosus* GG: A review. J Clin Gastroenterol. (2019) 53 Suppl 1: S1–s41. 10.1097/MCG.0000000000001170 30741841

[172] Szajewska H Kołodziej M. Systematic review with meta-analysis: *Lactobacillus rhamnosus* GG in the prevention of antibiotic-associated diarrhoea in children and adults. Alimentary Pharmacol Therap. (2015) 42:1149–57. 10.1111/apt.13404 26365389

[173] Korpela K Salonen A Virta LJ Kumpu M Kekkonen RA de Vos WM. *Lactobacillus rhamnosus* GG intake modifies preschool children's intestinal microbiota, alleviates penicillin-associated changes, and reduces antibiotic use. PLoS ONE. (2016) 11:e0154012. 10.1371/journal.pone.0154012 27111772 PMC4844131

[174] Yeung C-Y Chiang Chiau J-S Chan W-T Jiang C-B Cheng M-L Liu H-L. *In vitro* prevention of *Salmonella* lipopolysaccharide-induced damages in epithelial barrier function by various *Lactobacillus* Strains. Gastroenterol Res Pract. (2013) 2013:1–6. 10.1155/2013/973209 23840201 PMC3690232

[175] De Keersmaecker SC Verhoeven TL Desair J Marchal K Vanderleyden J Nagy I. Strong antimicrobial activity of *Lactobacillus rhamnosus* GG against *Salmonella typhimurium* is due to accumulation of lactic acid. FEMS Microbiol Lett. (2006) 259:89–96. 10.1111/j.1574-6968.2006.00250.x 16684107

[176] Liu J Gu Z Song F Zhang H Zhao J Chen W. Lactobacillus plantarum ZS2058 and *Lactobacillus rhamnosus* GG Use different mechanisms to prevent salmonella infection *in vivo*. Front Microbiol. (2019) 10:299. 10.3389/fmicb.2019.00299 30842764 PMC6391337

[177] Urcan AC Criste AD Dezmirean DS Bobiş O Victorit, a B Dulf FV. Botanical origin approach for a better understanding of chemical and nutritional composition of beebread as an important value-added food supplement. LWT. (2021) 2021:111068. 10.1016/j.lwt.2021.111068

[178] Pełka K Otłowska O Worobo RW Szweda P. Bee bread exhibits higher antimicrobial potential compared to bee pollen. Antibiotics. (2021) 10:125. 10.3390/antibiotics10020125 33525690 PMC7911093

[179] Pełka K Worobo RW Walkusz J Szweda P. Bee pollen and bee bread as a source of bacteria producing antimicrobials. Antibiotics. (2021) 10:713. 10.3390/antibiotics10060713 34199247 PMC8231920

[180] Kim KS. Current concepts on the pathogenesis of *Escherichia coli* meningitis: implications for therapy and prevention. Curr Opin Infect Dis. (2012) 25:273–8. 10.1097/QCO.0b013e3283521eb0 22395761

[181] He X Zeng Q Puthiyakunnon S Zeng Z Yang W Qiu J. *Lactobacillus rhamnosus* GG supernatant enhance neonatal resistance to systemic *Escherichia coli* K1 infection by accelerating development of intestinal defense. Sci Rep. (2017) 7:43305. 10.1038/srep43305 28262688 PMC5338013

[182] Harata G He F Hiruta N Kawase M Kubota A Hiramatsu M. Intranasal administration of *Lactobacillus rhamnosus* GG protects mice from H1N1 influenza virus infection by regulating respiratory immune responses. Lett Appl Microbiol. (2010) 50:597–602. 10.1111/j.1472-765X.2010.02844.x 20438620

[183] Ispirli H Dertli E. Detection of fructophilic lactic acid bacteria (FLAB) in bee bread and bee pollen samples and determination of their functional roles. J Food Process Preserv. (2021) 45:e15414. 10.1111/jfpp.15414

[184] Premaor MO Compston JE. The hidden burden of fractures in people living with HIV. JBMR Plus. (2018) 2:247–56. 10.1002/jbm4.10055 30283906 PMC6139727

[185] Post FA Hamzah L Fox J. Tenofovir disoproxil fumarate-associated bone loss: does vitamin D-binding protein play a role? AIDS. (2017) 31:178–9. 10.1097/QAD.0000000000001288 27898597

[186] Ohlsson C Sjögren K. Effects of the gut microbiota on bone mass. Trends Endocrinol Metab. (2015) 26:69–74. 10.1016/j.tem.2014.11.004 25497348

[187] Liu H Gu R Li W Zhou W Cong Z Xue J. *Lactobacillus rhamnosus* GG attenuates tenofovir disoproxil fumarate-induced bone loss in male mice via gut-microbiota-dependent anti-inflammation. Therap Adv Chronic Dis. (2019) 10:2040622319860653. 10.1177/2040622319860653 31321013 PMC6610433

[188] Gatej SM Bright R Weyrich LS Marino V Christophersen CT Gibson RJ. Probiotic *Lactobacillus Rhamnosus* GG Protects Against P. *Gingivalis* and *F. Nucleatum* gut dysbiosis. J Int Acad Periodontol. (2020) 22:18–27. 32224547

[189] Sapra L Dar HY Bhardwaj A Pandey A Kumari S Azam Z. *Lactobacillus rhamnosus* attenuates bone loss and maintains bone health by skewing Treg-Th17 cell balance in Ovx mice. Sci Rep. (2021) 11:80536. 10.1038/s41598-020-80536-2 33469043 PMC7815799

[190] Zhao H Zhao C Dong Y Zhang M Wang Y Li F. Inhibition of miR122a by *Lactobacillus rhamnosus* GG culture supernatant increases intestinal occludin expression and protects mice from alcoholic liver disease. Toxicol Lett. (2015) 234:194–200. 10.1016/j.toxlet.2015.03.002 25746479

[191] Liu Y Chen K Li F Gu Z Liu Q He L. Probiotic *Lactobacillus rhamnosus* GG prevents liver fibrosis through inhibiting hepatic bile acid synthesis and enhancing bile acid excretion in mice. Hepatology (Baltimore, Md). (2020) 71:2050–66. 10.1002/hep.30975 31571251 PMC7317518

[192] Owens JA Saeedi BJ Naudin CR Hunter-Chang S Barbian ME Eboka RU. *Lactobacillus rhamnosus* GG orchestrates an antitumor immune response. Cell Mol Gastroenterol Hepatol. (2021) 12:1311–27. 10.1016/j.jcmgh.2021.06.001 34111601 PMC8463873

[193] Oksaharju A. Probiotic *Lactobacillus rhamnosus* downregulates FCER1 and HRH4 expression in human mast cells. World J Gastroenterol. (2011) 17:750. 10.3748/wjg.v17.i6.750 21390145 PMC3042653

[194] Goyal N Shukla G. Probiotic *Lactobacillus rhamnosus* GG modulates the mucosal immune response in *Giardia intestinalis*-infected BALB/c mice. Digest Dis Sci. (2013) 58:1218–25. 10.1007/s10620-012-2503-y 23263901

[195] Menezes C Paludo CR Pupo MT. A review of the artificial diets used as pot-pollen substitutes. Pot-Pollen Stingless Bee Melittol. (2018) 2018:253–62. 10.1007/978-3-319-61839-5_18

[196] Ouwehand AC. Antiallergic effects of probiotics. J Nutr. (2007) 137: 794S−7S. 10.1093/jn/137.3.794S 17311977

[197] Blümer N Sel S Virna S Patrascan CC Zimmermann S Herz U. Perinatal maternal application of *Lactobacillus rhamnosus* GG suppresses allergic airway inflammation in mouse offspring. Clin Experi Allergy. (2007) 37:348–57. 10.1111/j.1365-2222.2007.02671.x 17359385

[198] Feleszko W Jaworska J Rha RD Steinhausen S Avagyan A Jaudszus A. Probiotic-induced suppression of allergic sensitization and airway inflammation is associated with an increase of T regulatory-dependent mechanisms in a murine model of asthma. Clin Experi Allergy. (2007) 37:498–505. 10.1111/j.1365-2222.2006.02629.x 17430345

[199] Thang CL Baurhoo B Boye JI Simpson BK Zhao X. Effects of *Lactobacillus rhamnosus* GG supplementation on cow's milk allergy in a mouse model. Allergy, Asthma Clin Immunol. (2011) 7:20. 10.1186/1710-1492-7-20 22145744 PMC3261804

[200] Kalliomäki M Salminen S Poussa T Arvilommi H Isolauri E. Probiotics and prevention of atopic disease: 4-year follow-up of a randomised placebo-controlled trial. Lancet. (2003) 361:1869–71. 10.1016/S0140-6736(03)13490-3 12788576

[201] Kukkonen K Savilahti E Haahtela T Juntunen-Backman K Korpela R Poussa T. Probiotics and prebiotic galacto-oligosaccharides in the prevention of allergic diseases: A randomized, double-blind, placebo-controlled trial. J Allergy Clin Immunol. (2007) 119:192–8. 10.1016/j.jaci.2006.09.009 17208601

[202] Simpson MR Dotterud CK Storrø O Johnsen R Øien T. Perinatal probiotic supplementation in the prevention of allergy related disease: 6 year follow up of a randomised controlled trial. BMC Dermatol. (2015) 15:13. 10.1186/s12895-015-0030-1 26232126 PMC4522068

[203] Wu CT Chen PJ Lee YT Ko JL Lue KH. Effects of immunomodulatory supplementation with *Lactobacillus rhamnosus* on airway inflammation in a mouse asthma model. J Microbiol Immunol Infect. (2016) 49:625–35. 10.1016/j.jmii.2014.08.001 25440975

[204] Wu CT Lin FH Lee YT Ku MS Lue KH. Effect of *Lactobacillus rhamnosus* GG immunopathologic changes in chronic mouse asthma model. J Microbiol Immunol Infect. (2019) 52:911–9. 10.1016/j.jmii.2019.03.002 30952512

[205] Kuitunen M Kukkonen K Juntunen-Backman K Korpela R Poussa T Tuure T. Probiotics prevent IgE-associated allergy until age 5 years in cesarean-delivered children but not in the total cohort. J Allergy Clin Immunol. (2009) 123:335–41. 10.1016/j.jaci.2008.11.019 19135235

[206] Betsi GI Papadavid E Falagas ME. Probiotics for the treatment or prevention of atopic dermatitis: a review of the evidence from randomized controlled trials. Am J Clin Dermatol. (2008) 9:93–103. 10.2165/00128071-200809020-00002 18284263

[207] Sepp E Julge K Vasar M Naaber P Björksten B Mikelsaar M. Intestinal microflora of Estonian and Swedish infants. Acta Paediatri. (1997) 86:956–61. 10.1111/j.1651-2227.1997.tb15178.x 9343275

[208] Kalliomäki M Salminen S Arvilommi H Kero P Koskinen P Isolauri E. Probiotics in primary prevention of atopic disease: a randomised placebo-controlled trial. Lancet (London, England). (2001) 357:1076–9. 10.1016/S0140-6736(00)04259-8 11297958

[209] Kalliomäki M Salminen S Poussa T Isolauri E. Probiotics during the first 7 years of life: a cumulative risk reduction of eczema in a randomized, placebo-controlled trial. J Allergy Clin Immunol. (2007) 119:1019–21. 10.1016/j.jaci.2006.12.608 17289135

[210] Rautava S Kalliomäki M Isolauri E. Probiotics during pregnancy and breast-feeding might confer immunomodulatory protection against atopic disease in the infant. J Allergy Clin Immunol. (2002) 109:119–21. 10.1067/mai.2002.120273 11799376

[211] Fölster-Holst R Müller F Schnopp N Abeck D Kreiselmaier I Lenz T. Prospective, randomized controlled trial on *Lactobacillus rhamnosus* in infants with moderate to severe atopic dermatitis. Br J Dermatol. (2006) 155:1256–61. 10.1111/j.1365-2133.2006.07558.x 17107398

[212] Sistek D Kelly R Wickens K Stanley T Fitzharris P Crane J. Is the effect of probiotics on atopic dermatitis confined to food sensitized children? Clin Experi Allergy. (2006) 36:629–33. 10.1111/j.1365-2222.2006.02485.x 16650048

[213] Boyle RJ Ismail IH Kivivuori S Licciardi PV Robins-Browne RM Mah LJ. *Lactobacillus* GG treatment during pregnancy for the prevention of eczema: a randomized controlled trial. Allergy. (2011) 66:509–16. 10.1111/j.1398-9995.2010.02507.x 21121927

[214] Brouwer ML Wolt-Plompen SA Dubois AE van der Heide S Jansen DF Hoijer MA. No effects of probiotics on atopic dermatitis in infancy: a randomized placebo-controlled trial. Clin Experi Allergy. (2006) 36:899–906. 10.1111/j.1365-2222.2006.02513.x 16839405

[215] Dotterud CK Storrø O Johnsen R Oien T. Probiotics in pregnant women to prevent allergic disease: a randomized, double-blind trial. Br J Dermatol. (2010) 163:616–23. 10.1111/j.1365-2133.2010.09889.x 20545688

[216] Filipovic I Ostojic O Vekovic V Lackovic M Zivkovic Z. Combination of *Lactobacillus Rhamnosus* LGG, Vitamin D3 and Zn in preventing atopic dermatitis in infancy. Am J Pediatrics. (2020) 6:273. 10.11648/j.ajp.20200603.26

[217] Vamanu A Vamanu E Popa O Campeanu G Albulescu R Drugulescu M. Obtaining of a symbiotic product based on lactic bacteria, pollen and honey. Pak J Biol Sci. (2008) 11:613–7. 18817135 10.3923/pjbs.2008.613.617

[218] Darby TM Naudin CR Luo L Jones RM. *Lactobacillus rhamnosus* GG-induced expression of leptin in the intestine orchestrates epithelial cell proliferation. Cell Mol Gastroenterol Hepatol. (2020) 9:627–39. 10.1016/j.jcmgh.2019.12.004 31874255 PMC7160578

[219] Fuenmayor CA Quicazán MC Figueroa J. Desarrollo De Un Suplemento Nutricional Mediante La Fermentación En Fase Sólida De Polen De Abejas Empleando Bacterias Ácido Lácticas Probióticas. (2011).

[220] Groah SL Rounds AK Ljungberg IH Sprague BM Frost JK Tractenberg RE. Intravesical *Lactobacillus rhamnosus* GG is safe and well tolerated in adults and children with neurogenic lower urinary tract dysfunction: first-in-human trial. Therap Adv Urol. (2019) 11:1756287219875594. 10.1177/1756287219875594 31620195 PMC6777056

[221] Forster CS Hsieh MH Pérez-Losada M Caldovic L Pohl H Ljungberg I. A single intravesical instillation of *Lactobacillus rhamnosus* GG is safe in children and adults with neuropathic bladder: A phase Ia clinical trial. J Spinal Cord Med. (2021) 44:62–9. 10.1080/10790268.2019.1616456 31100050 PMC7919893

[222] Hasler CM. Functional foods: benefits, concerns and challenges—a position paper from the American council on science and health. J Nutr. (2002) 132:3772–81. 10.1093/jn/132.12.3772 12468622

[223] European Union. Honey. Detailed Information on Honey Production, National Apiculture Programmes, Budget and Legal Bases. (2021).

[224] Capozzi V Fragasso M Russo P. Microbiological safety and the management of microbial resources in artisanal foods and beverages: the need for a transdisciplinary assessment to conciliate actual trends and risks avoidance. Microorganisms. (2020) 8:20306. 10.3390/microorganisms8020306 32098373 PMC7074853

[225] Marshall E Mejia D. Traditional Fermented Food and Beverages for Improved Livelihoods. Rome: FAO Diversification booklet; Rural Infrastructure and Agro-Industries Division, Food and Agriculture Organization of the United Nations (2011).

[226] Melini F Melini V Luziatelli F Ficca AG Ruzzi M. Health-promoting components in fermented foods: an up-to-date systematic review. Nutrients. (2019) 11:1189. 10.3390/nu11051189 31137859 PMC6567126

[227] Capozzi V Fragasso M Romaniello R Berbegal C Russo P Spano G. Spontaneous food fermentations and potential risks for human health. Fermentation. (2017) 3:49. 10.3390/fermentation3040049

[228] Tamang JP Watanabe K Holzapfel WH. Review: diversity of microorganisms in global fermented foods and beverages. Front Microbiol. (2016) 7:377. 10.3389/fmicb.2016.00377 27047484 PMC4805592

[229] Berbegal C Garofalo C Russo P Pati S Capozzi V Spano G. Use of autochthonous yeasts and bacteria in order to control brettanomyces bruxellensis in wine. Fermentation. (2017) 3:65. 10.3390/fermentation3040065

[230] Thakur M Nanda V. Composition and functionality of bee pollen: A review. Trends Food Sci Technol. (2020) 71:170–80. 10.1016/j.tifs.2020.02.001

